# Aurora kinase inhibition sensitizes melanoma cells to T-cell-mediated cytotoxicity

**DOI:** 10.1007/s00262-020-02748-9

**Published:** 2020-10-29

**Authors:** Simone Punt, Shruti Malu, Jodi A. McKenzie, Soraya Zorro Manrique, Elien M. Doorduijn, Rina M. Mbofung, Leila Williams, Deborah A. Silverman, Emily L. Ashkin, Ana Lucía Dominguez, Zhe Wang, Jie Qing Chen, Sourindra N. Maiti, Trang N. Tieu, Chengwen Liu, Chunyu Xu, Marie-Andrée Forget, Cara Haymaker, Jahan S. Khalili, Nikunj Satani, Florian Muller, Laurence J. N. Cooper, Willem W. Overwijk, Rodabe N. Amaria, Chantale Bernatchez, Timothy P. Heffernan, Weiyi Peng, Jason Roszik, Patrick Hwu

**Affiliations:** 1 Department of Melanoma Medical Oncology, The University of Texas MD Anderson Cancer Center, 1515 Holcombe Boulevard, Houston, TX 77030 USA; 2 Department of Pediatrics, The University of Texas MD Anderson Cancer Center, 1515 Holcombe Boulevard, Houston, TX 77030 USA; 3 Institute for Applied Cancer Science, The University of Texas MD Anderson Cancer Center, 1515 Holcombe Boulevard, Houston, TX 77030 USA; 4 Department of Cancer Systems Imaging, The University of Texas MD Anderson Cancer Center, 1515 Holcombe Boulevard, Houston, TX 77030 USA; 5 Department of Genomic Medicine, The University of Texas MD Anderson Cancer Center, 1515 Holcombe Boulevard, Houston, TX 77030 USA; 6 Sarcoma Medical Oncology, The University of Texas MD Anderson Cancer Center, 1515 Holcombe Boulevard, Houston, TX 77030 USA; 7 Division of Cancer Medicine, The University of Texas MD Anderson Cancer Center, Houston, TX USA; 8 Present Address: Immunitas Therapeutics, Cambridge, MA USA; 9 Present Address: Eisai Inc., Woodcliff Lake, NJ USA; 10 Present Address: Merck Research Laboratories, Palo Alto, CA USA; 11 Present Address: KSQ Therapeutics Inc., Cambridge, MA USA; 12 Present Address: Nature Cell Biology, Springer Nature, Shanghai City, China; 13 Present Address: EMD Serono, Rockland, MA USA; 14 Present Address: C4 Therapeutics, Watertown, MA USA; 15 Present Address: University of Houston, Houston, TX USA; 16 Present Address: SystImmune Inc., Redmond, WA USA; 17 Present Address: ZIOPHARM Oncology Inc., Boston, MA USA; 18 Present Address: Nektar Therapeutics, San Francisco, CA USA

**Keywords:** Aurora kinase, Melanoma, Immunotherapy, High-throughput screen, Immune checkpoint blockade, T-cell cytotoxicity

## Abstract

Although immunotherapy has achieved impressive durable clinical responses, many cancers respond only temporarily or not at all to immunotherapy. To find novel, targetable mechanisms of resistance to immunotherapy, patient-derived melanoma cell lines were transduced with 576 open reading frames, or exposed to arrayed libraries of 850 bioactive compounds, prior to co-culture with autologous tumor-infiltrating lymphocytes (TILs). The synergy between the targets and TILs to induce apoptosis, and the mechanisms of inhibiting resistance to TILs were interrogated. Gene expression analyses were performed on tumor samples from patients undergoing immunotherapy for metastatic melanoma. Finally, the effect of inhibiting the top targets on the efficacy of immunotherapy was investigated in multiple preclinical models. Aurora kinase was identified as a mediator of melanoma cell resistance to T-cell-mediated cytotoxicity in both complementary screens. Aurora kinase inhibitors were validated to synergize with T-cell-mediated cytotoxicity in vitro. The Aurora kinase inhibition-mediated sensitivity to T-cell cytotoxicity was shown to be partially driven by p21-mediated induction of cellular senescence. The expression levels of Aurora kinase and related proteins were inversely correlated with immune infiltration, response to immunotherapy and survival in melanoma patients. Aurora kinase inhibition showed variable responses in combination with immunotherapy in vivo, suggesting its activity is modified by other factors in the tumor microenvironment. These data suggest that Aurora kinase inhibition enhances T-cell cytotoxicity in vitro and can potentiate antitumor immunity in vivo in some but not all settings. Further studies are required to determine the mechanism of primary resistance to this therapeutic intervention.

**Electronic supplementary material:**

The online version of this article (10.1007/s00262-020-02748-9) contains supplementary material, which is available to authorized users.

## Introduction

Immunotherapy is able to induce durable disease control in a subset of patients with various cancer types, including metastatic melanoma. Adoptive cell therapy (ACT) using autologous tumor-infiltrating lymphocytes (TILs) has been associated with a predominantly durable objective response rate of around 50% in metastatic melanoma patients [1, 2], while immune checkpoint-blocking antibodies targeting cytotoxic T-lymphocyte–associated antigen 4 (CTLA4) and programmed cell death protein 1 (PD-1) have been associated with objective response rates of 10–20% (for anti-CTLA4), 30–45% (for anti-PD-1) and 50–60% for the anti-CTLA4/PD-1 combination [3, 4]. However, many tumors do not respond or become resistant to immunotherapy. In addition, the anti-CTLA4/PD-1 combination is associated with more frequent immune-related adverse events than either therapy alone, so improved therapy options are urgently needed.

In addition to immunotherapy, kinase inhibitors targeting BRAF and MEK are currently standard-of-care options for metastatic melanoma patients with confirmed BRAF V600 mutations [5]. A variety of targeted therapies are being investigated for their potential to increase tumor cell sensitivity to T-cell-mediated cytotoxicity [6], to achieve both the high response rates possible with targeted therapies and the often more durable clinical responses to immunotherapy. In the current study, we aimed to identify novel mechanisms of tumor-intrinsic resistance to T-cell-mediated cytotoxicity and increase tumor cell sensitivity to cancer immunotherapy by inhibiting these targets. We used two complementary high-throughput in vitro screens to identify targets that induce resistance to T-cell-mediated cytotoxicity. Both open reading frame (ORF) and compound screening independently identified Aurora kinase B (AURKB) as a resistance gene with regard to immunotherapy. The synergy between Aurora kinase inhibitors (AURKi) and immunotherapy was investigated using in vitro and in vivo assays.

## Methods

### Patient-derived melanoma samples and cell lines

The human melanoma cell lines Mel2338, Mel2549, Mel2559, Mel2686, and Mel2812 and their autologous TILs were derived from tumors of metastatic melanoma patients at The University of Texas MD Anderson Cancer Center using an Institutional Review Board-approved laboratory protocol (LAB06-0755) as previously described [7]. All melanoma cell lines were maintained in RPMI 1640 (Thermo Fisher Scientific) supplemented with heat-inactivated fetal bovine serum (10% FBS, Gemini Bio Products), HEPES (10 mM, Corning), GlutaMAX-I, Insulin-Transferrin-Selenium, 2-Mercaptoethanol (55 µM; all from Thermo Fisher Scientific), and Normocin (100 µg/ml, InvivoGen). All cell lines were verified by short tandem repeat fingerprinting or matching mutational profiles, kept at low passage numbers, and routinely tested for mycoplasma. TILs were generated as previously described [8]. For NanoString analyses, sufficient RNA was isolated from formalin-fixed, paraffin-embedded tumor samples obtained from 23 patients prior to receiving TIL ACT. Among these 23 patients, 10 had a partial (*n* = 7) or complete (*n* = 3) response, and were categorized as responders to TIL ACT, and 13 had stable (*n* = 8) or progressive disease (*n* = 5), and were categorized as nonresponders to TIL ACT. Patient samples were handled according to the medical ethical guidelines described in the Declaration of Helsinki.

### High-throughput ORF screen

Mel2549 cells were transduced with an arrayed library of 576 ORF-expressing lentiviruses by spin infection in the presence of polybrene (4 µg/ml) and assayed for sensitivity to autologous TILs (supplementary figure 1a) as described before [9]. Of the 576 ORFs, 384 are kinases and 192 are involved in epigenetic regulation of gene expression (collated and graciously shared by Dr. R. DePinho, MD Anderson Cancer Center). For validation purposes, the individual ORFs were transduced in human melanoma cell lines in a 6-well plate by spin infection in the presence of 8 µg/ml polybrene.

### High-throughput compound screen

A library of 850 bioactive compounds (Selleckchem) was screened for synergy with autologous TIL-mediated cytotoxicity in Mel2338 and Mel2549 melanoma cell lines (supplementary figure 1b) as described previously [10]. Briefly, melanoma cells were treated with 1 µM of compound for 24 h in triplicate and, after washing off drugs with PBS, challenged with autologous tumor-reactive TILs or control medium for three hours. A cleaved caspase-3 cytotoxicity assay was performed to assess tumor cell apoptosis.

### Cleaved caspase-3 cytotoxicity assay and comboscore calculation

The cleaved caspase-3 cytotoxicity assay was performed as previously described [11]. Briefly, melanoma cells were stained for intracellular cleaved caspase-3, followed by flow cytometry analysis. Based on the percentage of cells positive for cleaved caspase-3, a comboscore was calculated to assess the increase in tumor cell apoptosis caused by the combination of T-cell cytotoxicity and genetic modification or drug treatment versus the modification or drug treatment alone, normalized for the apoptosis induced by T cells and control treatment. The comboscore, in which a drug can be replaced by ORF, was obtained using the following formula [12]:

$${\text{Comboscore}} = \left( {\frac{{\left( {\% {\text{ caspase}} + {\text{tumor cells}}){\text{drug}} + {\text{T cells}} - (\% {\text{caspase}} + {\text{tumor cells}}} \right){\text{drug}}}}{{\left( {\% {\text{caspase}} + {\text{tumor cells}}){\text{control}} + {\text{T cells}} - (\% {\text{caspase}} + {\text{tumor cells}}} \right){\text{control}}}}} \right)$$

A modification that enhances tumor cell sensitivity to T-cell-mediated cytotoxicity induces a comboscore greater than 1.

### Analysis of senescence phenotype

Mel2549 and Mel2812 cells were transfected with p21 siRNA or control siRNA (80 pmol per 2 × 10^5^ seeded cells, Santa Cruz Biotechnology) overnight. The next day, transfected cells were seeded in a 96-well plate (5 × 10^4^ cells/well) and treated with AZD1152 (barasertib, 2 µM, Selleck Chemicals) or DMSO for 24 h. As a positive control for senescence induction, cells were treated with H_2_O_2_ (150 µM) for 2 h, before replacing the H_2_O_2_-containing medium with regular culture medium. Cells were challenged with TILs 24 h after transfection and stained for intracellular cleaved caspase-3. All conditions were tested at least in triplicate. For Western blot and β-galactosidase analysis, cells were kept in culture in the presence of compounds for four days after siRNA treatment. Pellets of at least 1 × 10^5^ treated cells were stored at − 80 °C. Simultaneously, equal cell numbers from various pretreatment conditions were seeded and stained for senescence-associated β-galactosidase using the Senescence β-Galactosidase Staining Kit according to the manufacturers’ instructions (#9860, Cell Signaling Technology). Four images per condition at similar random positions throughout the wells were acquired for analysis using an Axiovert 200 microscope equipped with A-Plan 10x/0.25 Ph1 and LD A-Plan 40x/0.50 Ph2 objectives (Zeiss).

### Western blot analyses

Cell pellets were lysed in RIPA lysis buffer (Santa Cruz Biotechnology) according to the manufacturer’s instructions. Cell lysate protein (25 µg) was separated in 4%-20% SDS polyacrylamide gel lanes and transferred to nitrocellulose membranes. Membranes were blocked in 5% milk or 5% bovine serum albumin in TBST and incubated with primary antibodies targeted against β-actin (#4970), p21 (#2947), phosphorylated histone H3 (#53348), phosphorylated Rb (#9301), or Rb (#9309) (all from Cell Signaling Technology). The membranes were then washed, incubated with secondary antirabbit (#7074) and antimouse (#7076) IgG antibodies tagged with horseradish peroxidase and developed using SignalFire ECL Reagent or SignalFire Plus ECL Reagent (all from Cell Signaling Technology). All samples were probed with different antibodies on the same membrane, always including β-actin loading control. Membranes were either cut, or stripped of antibodies using Restore PLUS Western Blot Stripping Buffer (Thermo Fisher Scientific). Densitometry was assessed using the ImageJ gel lane area calculation tool (ImageJ version 1.50i; https://imagej.nih.gov/ij).

### Expression analyses in patient-derived melanoma samples

The Cancer Genome Atlas (TCGA, https://cancergenome.nih.gov) SKCM dataset was studied for the correlations between *AURKA* and *AURKB* mRNA expression and overall survival. Total RNA was isolated from five 10 μm formalin-fixed, paraffin-embedded sections from 23 melanoma samples using the AllPrep DNA/RNA FFPE kit (QIAGEN) according to the manufacturers’ instructions. Melanin was removed using the Zymogen OneStep PCR Inhibitor Removal Kit (Zymo Research). A panel of 30 custom NanoString probes (NanoString™ Technologies) was prepared, including the genes that received the lowest comboscore in the ORF screen and genes implicated in the function of Aurora kinases. RNA (400 ng) was hybridized to the probes and subjected to NanoString nCounter analysis according to the manufacturers’ instructions. We also used two publicly available RNA sequencing datasets: a dataset including 27 melanoma samples from patients who received anti-PD-1 therapy (26 pretreatment and one early on-treatment; only the first of two samples derived from the same patient was included) [13] and a dataset including 24 melanoma samples from patients who received anti-CTLA4 therapy (9 pre and 15 post-treatment initiation) [14].

### Murine cells and models

The MC38/gp100 cell line was established as described previously [15]. B16 cells were obtained from the National Cancer Institute. The BP cell line was established as described previously [16]. MC38/gp100, B16, and BP cells were all maintained in the culture media described above for human melanoma cell lines, excluding the Insulin–Transferrin–Selenium supplement. For RNAseq analysis, 1.0 × 10^6^ cells were plated in 6-well plates, detached with trypsin after 24 h, washed once with culture medium and twice with PBS, resuspended in 1 ml RNAlater and submitted for sequencing analysis. The D4M UV2 cell line was kindly provided by Dr. David E. Fisher, Massachusetts General Hospital and maintained in DMEM 11965–092 (Thermo Fisher Scientific) supplemented with 10% FBS, GlutaMAX-I, 2-mercaptoethanol and penicillin/streptomycin. Thy1.1^+^ Pmel-1 transgenic mice (harboring a gp100-specific TCR) were kindly provided by Dr. Nicholas Restifo (Surgery Branch, National Cancer Institute, Bethesda, MD). Six- to twelve-week-old female C57BL/6 mice (Charles River, Frederick Research Model Facility) were inoculated subcutaneously with 0.5 × 10^6^ tumor cells on day 0. Mice were treated with AZD1152 (25 mg/kg) on days 3–6; anti-CTLA4 (100 µg, clone 9H10, Bio X Cell) on days 3, 6, 9, and 15; the combination; or vehicle plus isotype control (*n* = 5–10 per group, performed twice), unless described otherwise. On day 16, MC38/gp100 tumors were harvested from three mice per group and the infiltrating immune cells were isolated and stained for CD3, CD4, CD8, CD25, CD45, and FoxP3 as described before [10]. After AZD1152 or vehicle treatment on days 5–8 intraperitoneally (i.p.) or on days 11–14 intratumorally (i.t.), tumors (*n* = 3/group) were harvested into RNAlater (Qiagen) the day following the final treatment and submitted for RNA extraction and RNAseq analysis. For combination treatment, Pmel-1 T cells were adoptively transferred seven days post B16 tumor cell inoculation, as described previously [15]. All mice were maintained in a pathogen-free barrier facility and handled in accordance with protocols approved by the Institutional Animal Care and Use Committee.

### Statistical analyses

Analyses for synergy between each compound and TIL treatment were performed using CalcuSyn software (Biosoft). The CalcuSyn software quantifies whether the effects of two agents are synergistic (combination index < 1), additive (combination index = 1) or antagonistic (combination index > 1) based on the Chou–Talalay method [17]. Two-sided independent sample *t* tests (for data following a normal distribution) or Mann–Whitney *U* tests (for data that did not follow a normal distribution) were performed to compare expression levels between responding and nonresponding patients and to compare stained cell fractions. Kruskal–Wallis nonparametric tests were used to compare gene expression levels among three cohorts. Repeated measures analysis of variance was used to compare tumor sizes between treatment groups in vivo. The effects on survival were analyzed using Kaplan–Meier curves and log-rank analysis. A *p* value below 0.05 was considered statistically significant. Graphs were generated using GraphPad Prism 6 (GraphPad Software) and Tableau (Tableau Software). Statistical analyses were performed using SPSS version 23 (IBM). Unless otherwise specified, the data are represented as mean ± standard error of the mean.

## Results

### Aurora kinase identified to mediate resistance to T-cell-mediated cytotoxicity

**Fig. 1:**
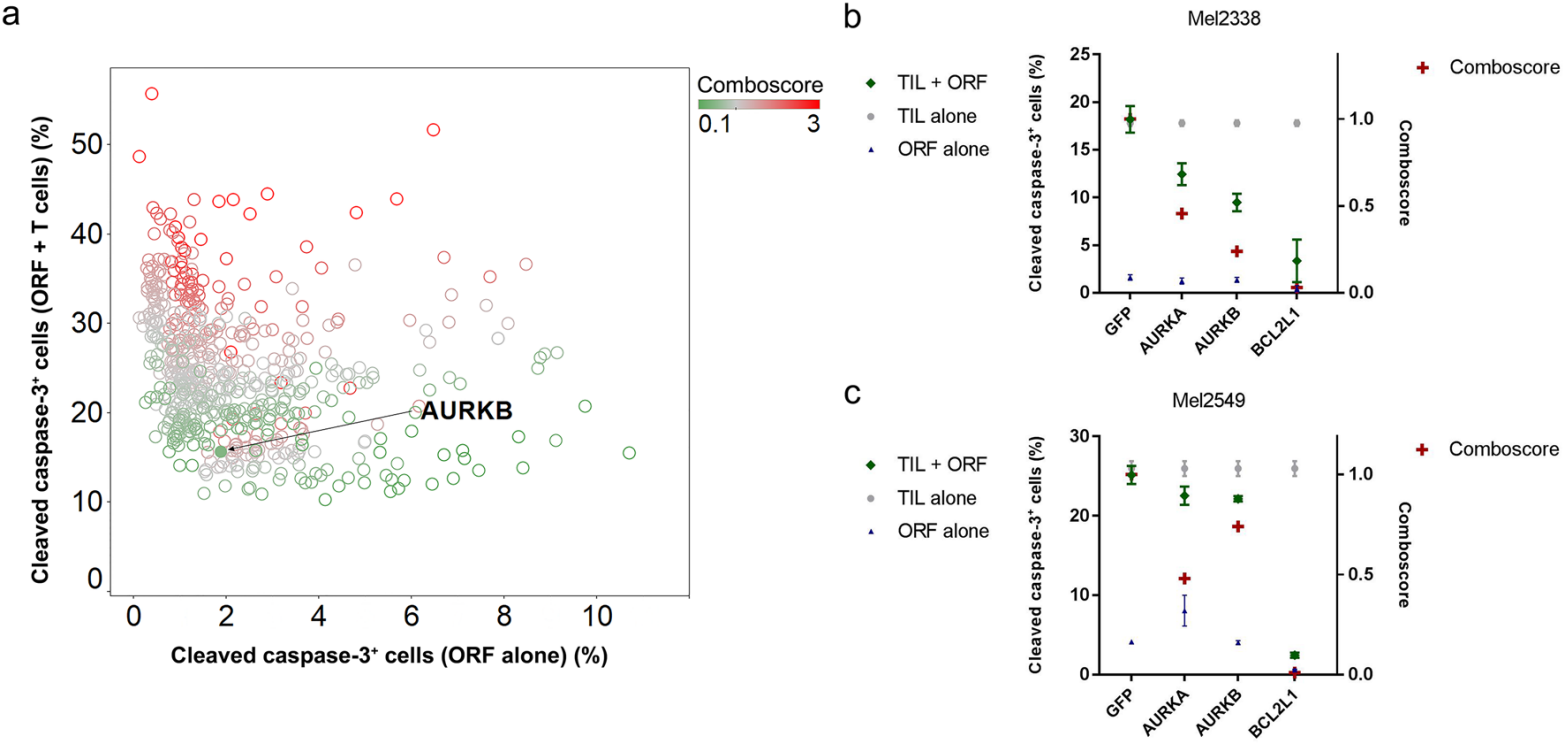
Aurora kinase overexpression induces resistance to T-cell-mediated cytotoxicity. **a** A high-throughput ORF screen showed that *AURKB* overexpression enhanced resistance to T-cell-mediated cytotoxicity. The cytotoxicity induced by expression of an ORF alone (x axis) versus cytotoxicity induced by the combination of an ORF and autologous T cells (y axis) in Mel2549 cells is shown. Each circle represents one ORF, while the color intensity represents the comboscore (a comboscore of 1, representing no additional effect of T-cell treatment, is shown in gray). The *AURKB* ORF is indicated by an arrow and solid circle (comboscore = 0.45). Overexpression of Aurora kinases A and B in Mel2338 (**b**) and Mel2549 (**c**) was confirmed to decrease comboscores compared with the GFP control. Comboscores ( +) were superimposed on the cleaved caspase-3 percentages induced by ORF alone (Δ), TIL alone (o), and ORF plus TIL (◊). *BCL2L1* overexpression is shown as a positive control for suppression of T-cell cytotoxicity

To identify genes whose expression by cancer cells can mediate escape from immune cell targeting, we performed an arrayed 576 ORF expression screen in melanoma cell lines to identify candidate genes that impart resistance to T-cell-mediated cytotoxicity (supplementary figure 1a) [9]. Through this approach, *AURKB* was identified among the genes with the lowest comboscores (Fig. 1a). Aurora kinase A (*AURKA*) was not part of the ORF library. As a complementary approach to identify compounds that can potentiate the efficacy of immunotherapy, we performed an in vitro screen of an 850 compound library, using matched pairs of patient-derived melanoma cancer cells and TILs as model system (supplementary figure 1b) [10]. Multiple inhibitors of Aurora kinases, enzymes involved in mitosis, enhanced the sensitivity of melanoma cells to T-cell-induced cytotoxicity: four AURKi induced increased comboscores in Mel2338 cells (supplementary figure 2a) and six AURKi in Mel2549 cells (supplementary figure 2b). These data suggest that Aurora kinase inhibition can enhance the efficacy of T-cell cytotoxicity.

### Aurora kinase inhibition validated to sensitize melanoma cells to T-cell-mediated cytotoxicity

**Fig. 2:**
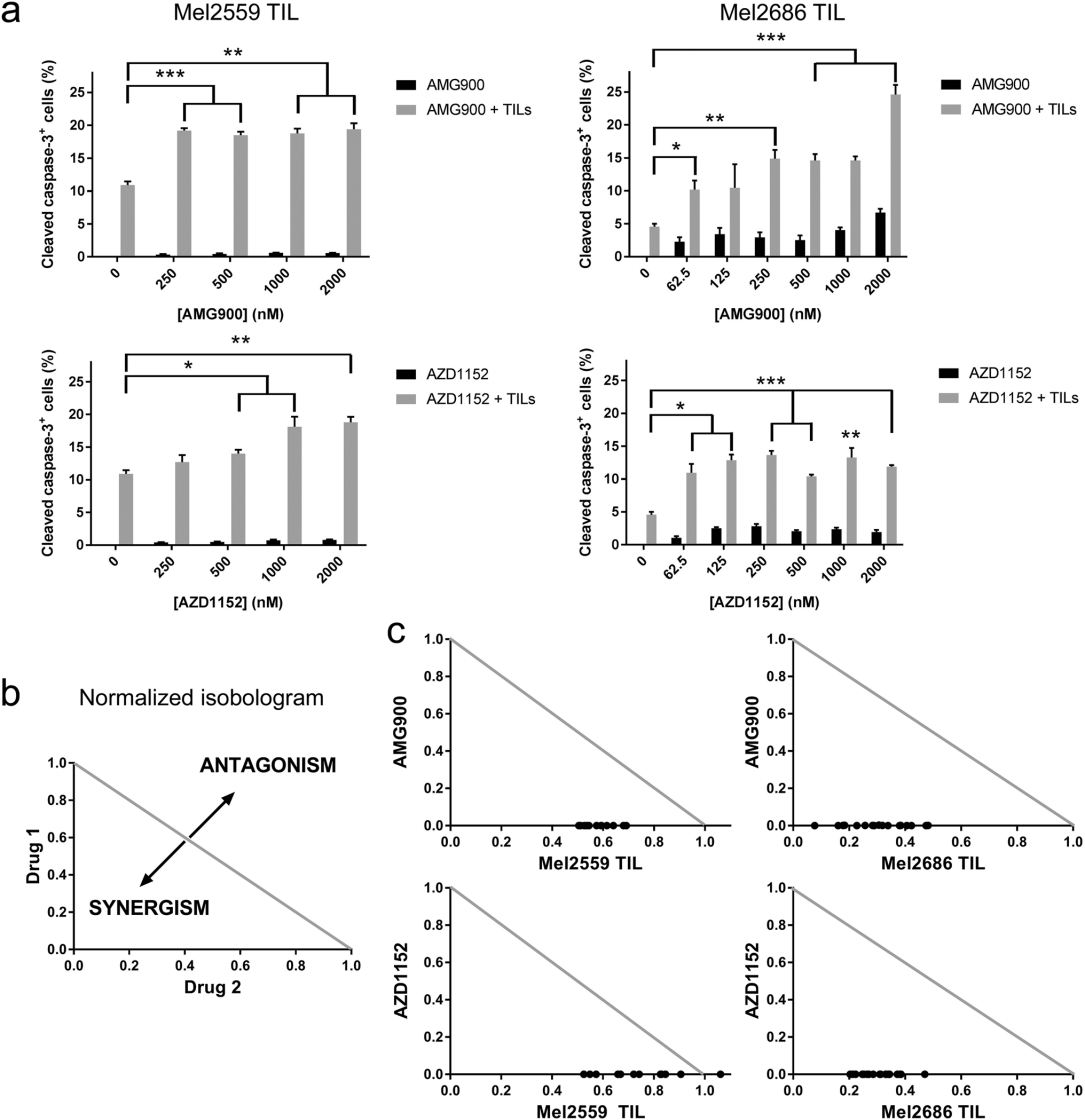
Aurora kinase inhibitors and TILs synergize in inducing apoptosis in human melanoma cells. **a** The human melanoma-derived cell lines Mel2559 and Mel2686 were treated with increasing concentrations of pan-Aurora kinase inhibitor AMG900 or AURKB inhibitor AZD1152, followed by co-culture with autologous TILs. The percentage of cleaved caspase-3 was subsequently analyzed to quantify apoptosis. **b** A combination index was calculated to quantify synergy between Aurora kinase inhibitors and melanoma-derived TILs using CalcuSyn. The normalized dose effect of each drug is represented on the representative axes. The combination index between the drugs is indicated in the graph by black dots, and the interaction is synergistic if the combination index is < 1, below the diagonal line. **c** The combination indices of AMG900 or AZD1152 with Mel2559 or Mel2686 TIL are represented in the normalized isobolograms. The data are representative of at least three independent experiments. Two-sided independent sample *t* tests were performed to compare cleaved caspase-3^+^ cell frequencies induced by a compound and TIL with TIL alone. *p < 0.05, **p < 0.01, ***p < 0.001

To validate that Aurora kinase induces resistance to T-cell-mediated cytotoxicity, *AURKA* and *AURKB* ORFs were stably transduced in Mel2549 and Mel2338 cells. After co-culture with autologous TILs, melanoma cells overexpressing *AURKA* or *AURKB* were more resistant to T-cell-mediated cytotoxicity, as evidenced by decreased comboscores (Fig. 1b, c). We then selected two AURKi for further analysis: pan-AURKi AMG900 and AURKB-specific inhibitor AZD1152. Both AURKi induced synergy with T-cell-mediated cytotoxicity in the four melanoma cell lines tested (Fig. 2, supplementary figure 3) and did not decrease the viability, proliferation rate or intrinsic cytotoxicity of TILs (supplementary figure 4). These results show that the inhibition of Aurora kinase can enhance TIL-mediated cytotoxicity.

### Mechanism of induced sensitivity: AURKi induce cellular senescence

**Fig. 3:**
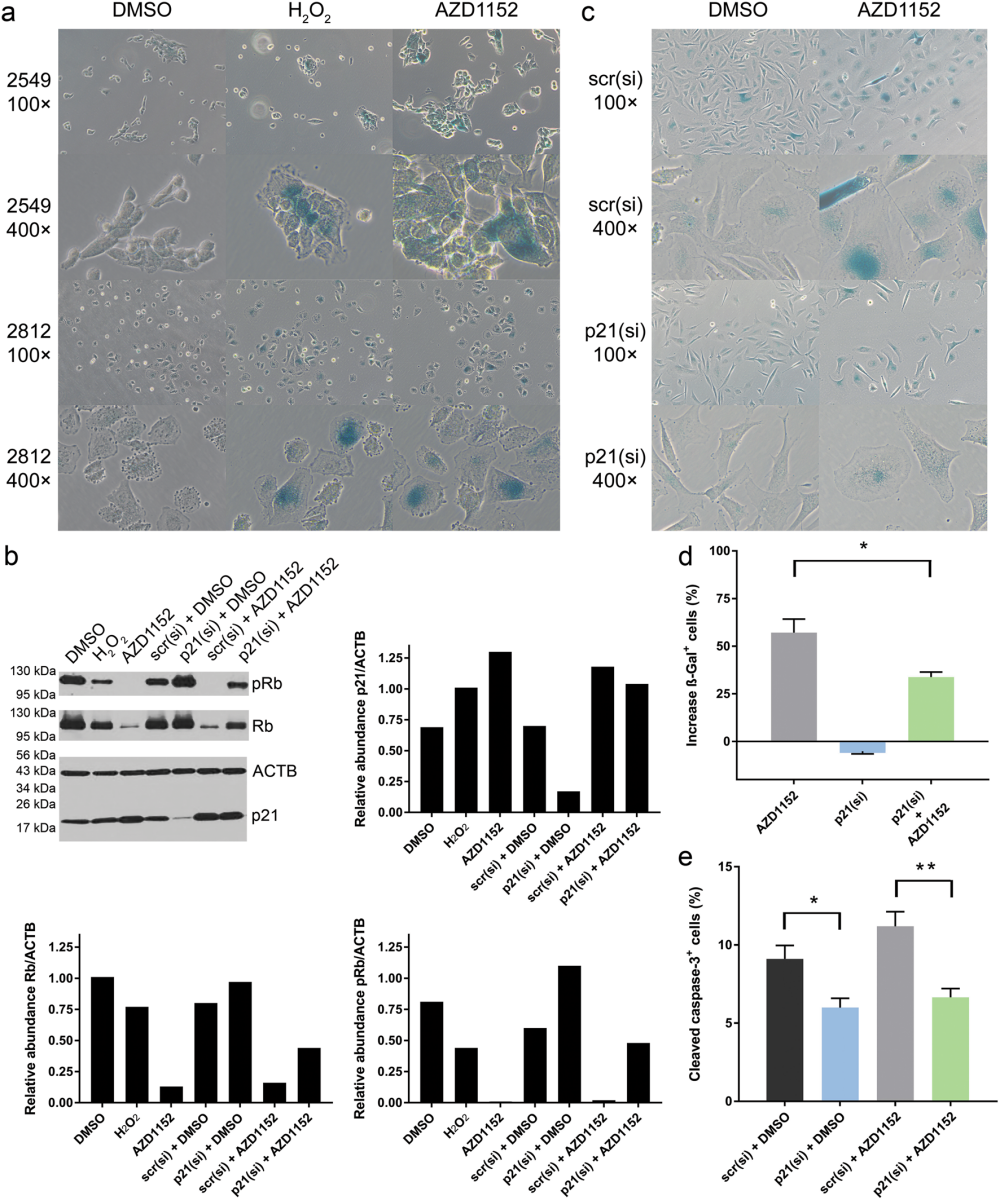
Aurora kinase inhibition induces cellular senescence. **a** Mel2549 and Mel2812 were treated with AZD1152 or DMSO control and stained for senescence marker β-galactosidase. Treatment with H_2_O_2_ was used as a positive control for senescence induction. **b** Western blot analysis of p21, pRb, and Rb expression after treatment of Mel2812 with various compound and siRNA combinations. ACTB was used as loading control. A densitometric analysis of the band intensity relative to the ACTB control is shown for the three proteins. **c** After treatment of Mel2812 with scrambled (scr(si)) or p21-targeting siRNA (p21(si)) combined with AZD1152 or DMSO, cells were stained for β-galactosidase. **d** The increase in positive cell fraction compared with cells treated with scrambled siRNA and DMSO in three experiments. **e** Flow cytometry-based analysis of the fraction of cleaved caspase-3^+^ cells after treatment of Mel2812 cells with a combination of scrambled or p21-targeting siRNA and AZD1152 or DMSO. Each imaging and flow cytometry-based cell quantification experiment was repeated at least three times with three to four biologic replicates. Differences in cell frequencies were analyzed by two-sided independent sample *t* tests. *p < 0.05. **p < 0.01

We tested whether AURKi directly enhance T-cell cytotoxicity by upregulating tumor cell expression of MHC class I and II, but this was not the case (supplementary figure. 5). On the basis of reports that Aurora kinases regulate not only cell cycle, DNA damage, and apoptosis but also autophagy and senescence [18, 19], which have been implicated as immune-sensitizing cellular states [12, 20, 21], we hypothesized that Aurora kinase inhibition enhances sensitivity to T-cell-mediated cytotoxicity by inducing tumor cell autophagy or senescence. First, we confirmed that the AURKi AZD1152 functionally blocks AURKB at the dose used in these experiments, using the absence of phosphorylation of its substrate histone H3 as a readout (supplementary figure. 6) [22]. We did not find consistent upregulation of autophagy markers LC3B and p62 after blocking Aurora kinase, but treating Mel2549 and Mel2812 with AZD1152 increased the levels of senescence-associated β-galactosidase, a marker for senescence [23] (Fig. 3a). Further studying the senescence pathway, we observed strong upregulation of p21 and downregulation of pRb, critical senescence mediators, in Mel2549 and Mel2812 treated with AZD1152 (Fig. 3b). Upregulation of p21 and downregulation of pRb could be partly prevented by co-inhibiting p21 using siRNA (Fig. 3b), which significantly reduced the senescence phenotype induced by the AURKi AZD1152 (*p* = 0.037; Fig. 3c, d). These data suggest that Aurora kinase inhibition at least partly induces cellular senescence through upregulation of p21. Induction of senescence by H_2_O_2_ [24] (Fig. 3a) increased Mel 2812 sensitivity to T-cell-induced cytotoxicity (supplementary figure. 7). As expected, p21 knockdown reduced sensitivity to TIL cytotoxicity, particularly when this sensitivity was enhanced by AZD1152 (*p* = 0.009; Fig. 3e). These data show that AURKB inhibition induces tumor cell senescence, enhancing sensitivity to T-cell cytotoxicity.

To confirm that AURKi-induced increased sensitivity to TIL was a tumor cell-intrinsic phenomenon and not merely a representation of decreased tumor cell numbers, we performed the TIL co-culture assay after seeding equal numbers of Mel2812 cells pretreated with drugs, showing that AZD1152 directly increased the tumor cell-intrinsic sensitivity to T-cell-induced cytotoxicity (supplementary figure. 8).

### High Aurora kinase expression is associated with resistance to Immunotherapy and poor survival in melanoma patients

Increased expression levels of *AURKA* [25] or *AURKB* are significantly correlated with poor patient survival in metastatic melanoma patients (supplementary figure. 9). Furthermore, *AURKA* was expressed at a significantly higher level in tumor samples from metastatic melanoma patients who did not respond to TIL ACT compared with those who responded (*p* = 0.042; supplementary figure. 10a). Transcripts of other proteins involved in the Aurora kinase pathway were also more abundant in tumors from nonresponding patients, including *AURKB* (*p* = 0.078) and *CDCA8* (*p* = 0.075), which encodes a protein that interacts with AURKB and forms part of the chromosomal passenger complex involved in cell division [26]. Statistical significance was not reached, potentially owing to the small sample size (*n* = 23). In an independent, publicly available RNA sequencing dataset of patient-derived melanoma samples, *AURKA*, *AURKB*, and *CDCA8* showed trends toward increased expression in patients who did not respond to anti-PD-1 therapy (progressive disease; supplementary figure. 10b). We analyzed the same correlations in a publicly available dataset of patient-derived melanoma samples obtained before or after initiation of anti-CTLA4 treatment. Correlations with response to anti-CTLA4 treatment were not statistically significant, potentially owing to small sample sizes. However, both *AURKA* and *AURKB* expression were significantly inversely correlated with markers for T-cell infiltration of the tumor: *CD3E*, *CD4*, *CD8A*, *LCK*, *PDCD1* and *IFNG* (Tables 1, 2). These data suggest that high Aurora kinase expression is associated with immune suppression and resistance of melanoma to T-cell-mediated immunotherapy.

**Table 1:** Inverse correlation between *AURKA* expression and T-cell infiltration markers

gene	*R*	*p* value
*CD3E*	− 0.53	0.007
*CD4*	− 0.50	0.012
*CD8A*	− 0.48	0.019
*LCK*	− 0.52	0.009
*PDCD1*	− 0.53	0.007
*IFNG*	− 0.43	0.036

The expression of *AURKA* is significantly inversely correlated with the expression of genes associated with T-cell infiltration of the tumor in a dataset derived from a human melanoma CTLA4 study [14]

*R* spearman correlation

**Table 2:** Inverse correlation between *AURKB* expression and T-cell infiltration markers

gene	*R*	*p* value
*CD3E*	− 0.35	0.096
*CD4*	− 0.31	0.134
*CD8A*	− 0.39	0.056
*LCK*	− 0.30	0.161
*PDCD1*	− 0.38	0.070
*IFNG*	− 0.41	0.045

The expression of *AURKB* is significantly inversely correlated with the expression of *IFNG* and shows a trend toward an inverse correlation with *CD8A* expression in a dataset derived from a human melanoma CTLA4 study [14]

*R* spearman correlation

### AURKi treatment significantly improves immunotherapy efficacy in some but not all preclinical melanoma models

**Fig. 4:**
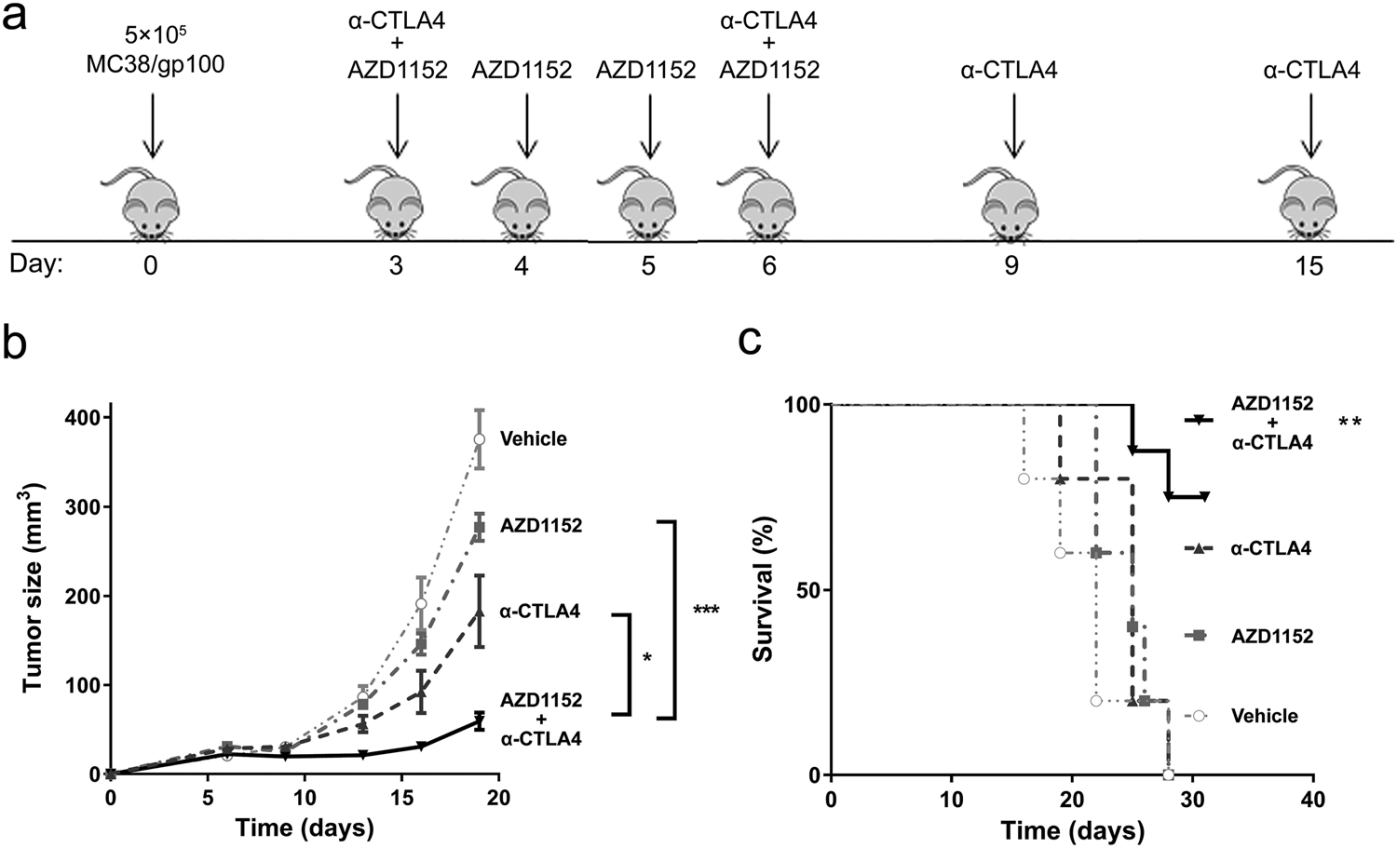
Aurora kinase B inhibitor enhanced the efficacy of immunotherapy in MC38/gp100 tumor model. **a** Regimen by which mice received the combination of 25 mg/kg Aurora kinase B inhibitor AZD1152 and 100 µg of anti-CTLA4 checkpoint blockade immunotherapy. **b** Although AZD1152 by itself did not significantly decrease tumor growth (*p* = 0.361), the combination of AZD1152 and anti-CTLA4 significantly reduced tumor growth compared with AZD1152 (*p* < 0.001) or anti-CTLA4 (*p* = 0.019) alone (Fig. 4b). Size differences between the groups were analyzed by repeated measures analysis of variance. **c** Kaplan–Meier curves and log-rank analysis showed that treatment with the combination of AZD1152 and anti-CTLA4 resulted in significantly improved survival (*p* = 0.002). *N* = 5 per group; the data are representative of two independent experiments: *p < 0.05; **p < 0.01; ***p < 0.001

To determine whether the synergy of AURKi with T-cell-mediated cytotoxicity identified in vitro and the inverse correlation between Aurora kinase expression and response to immunotherapy in patient samples could be translated to an in vivo potentiation of immunotherapy, we studied the efficacy of concurrent AURKi treatment and T-cell checkpoint blockade in murine cancer models. AURKB inhibitor AZD1152 was combined with anti-CTLA4 to treat MC38/gp100 tumors in syngeneic mice (Fig. 4a). The combination treatment resulted in significantly reduced tumor growth compared with AZD1152 (*p* < 0.001) or anti-CTLA4 (*p* = 0.019) alone (Fig. 4b) and significantly improved survival (*p* = 0.002; Fig. 4c). Tumor-infiltrating CD4^+^ or CD8^+^ T-cell frequency was not affected by AURKi treatment (supplementary figure. 11). When this treatment was repeated with larger tumors (tumor size ≥ 15 mm^2^ at treatment initiation), the combination therapy did not improve anti-CTLA4 efficacy (supplementary figure. 12a). Similarly, no improved efficacy of anti-CTLA4 by AZD1152 was observed in BP or D4M-UV2 melanoma models (supplementary figure. 12b,c). AZD1152 treatment did not improve tumor control induced by anti-PD1 treatment in the MC38/gp100 or D4M UV2 model either (supplementary figure. 13). Because B16 melanoma is poorly responsive to checkpoint blockade, we investigated the effect of combining AZD1152 treatment with Pmel-1 ACT. The combination therapy showed a marginal trend toward improved efficacy, both when the drug was administered i.p. or i.t. (supplementary figure. 14). *Aurka* and *Aurkb* were expressed at similar levels in B16 (101 and 111 TPM), BP (103 and 111 TPM) and MC38/gp100 (122 and 141 TPM) cells in vitro. RNAseq analysis of B16 tumors confirmed that the expression of *Hist2h3c1*, a predominant AURKB target, was significantly reduced following both i.p. (FC = 0.13, *p* = 0.017) and i.t. (FC = 0.047, *p* < 0.0001) AZD1152 treatment compared to vehicle treatment, indicating that the drug was appropriately targeting AURKB in the tumor microenvironment. When AZD1152 treatment was initiated later, the marginal difference in tumor growth was lost. These data suggest that Aurora kinase blockade can enhance the efficacy of immunotherapy in vivo in small tumors, but may be insufficient to improve immunotherapy efficacy in more established tumors.

## Discussion

A high-throughput ORF screen identified *AURKB* overexpression to mediate resistance to T-cell-mediated cytotoxicity, and both *AURKA* and *AURKB* overexpression were separately validated to induce resistance to T-cell cytotoxicity. Using a complementary high-throughput 850 compounds screen, multiple AURKi obtained high synergy with TIL treatment to induce tumor cell apoptosis. Two AURKi were independently validated to induce synergy with T-cell-mediated cytotoxicity in multiple melanoma cell lines. These data indicate that Aurora kinase inhibition may potentiate T-cell-based immunotherapy by increasing sensitivity to T-cell-mediated cytotoxicity. Indeed, the efficacy of AURKi treatment has been suggested to be dependent on the immune response [27].

Aurora kinases are serine/threonine protein kinases that regulate mitosis. AURKA regulates mitotic entry, chromosome segregation, and genomic stability [28, 29]. Inhibition of AURKA leads to cell cycle arrest, polyploidy, and apoptosis. AURKB is essential for chromosome biorientation and attachment [30]. AURKB depletion causes absence of the mitotic checkpoint and cytokinesis, resulting in polyploidy as well [28], specifically tetraploid senescence [18]. Aurora kinases have gained attention owing to their broad functions in cell cycle, DNA damage response, and apoptosis [28, 31], as well as their upregulation in various types of cancer and the inverse correlation between Aurora kinase expression and prognosis [32, 33]. Pan-AURKi AMG900 is one of the furthest developed AURKi, showing promising preclinical [34] and clinical [35] activity. AMG900 has also been shown to induce senescence in glioblastoma cells [36]. AZD1152 (barasertib) has been shown to selectively inhibit AURKB and inhibit tumor growth in a dose-dependent manner in different preclinical xenograft models [34]. Owing to limited response rates and adverse effects attributed to AURKi treatment [37], the efficacy of AURKi in combination with other targeted therapies and chemotherapy is being investigated [38, 39].

Because Aurora kinases are involved in many signaling pathways, we sought to identify the mechanism by which AURKi increased sensitivity to T-cell-mediated cytotoxicity. AURKB inhibition by AZD1152 was found to induce a predominantly senescent phenotype. Accordingly, AURKA inhibitor MLN8237, AURKB inhibitor AZD1152 and pan-AURKi VX-680 have previously been shown to induce a senescence phenotype and sensitize the cells to apoptosis induced by death receptor activation [40]. As Aurora kinases have a crucial function in mitosis, their inhibition typically leads to halted cell proliferation, DNA damage, and potentially a senescent phenotype. By blocking cytokinesis, AURKi can induce a state that is not immediately cytotoxic in itself but may slow progression of disease rather than produce complete response, which is consistent with clinical trial data [37]. In addition, this state can sensitize tumor cells to T-cell-based immunotherapy, as T cells have been shown to clear senescent cells [41]. We found that this sensitization to T-cell-mediated cytotoxicity indeed occurred for AZD1152-treated cells and that this could be partially reverted by blocking p21 expression. This finding corresponds with the findings of an earlier study by Fitzner et al., which also showed that induction of the senescence phenotype could be blocked by p21 siRNA [42]. Aurora kinase inhibition was thus shown to increase the immunogenicity of tumor cells, one of the pathways important for resistance to immunotherapy [43], at least partially by p21-mediated induction of a senescence phenotype.

Using a set of melanoma samples derived from patients undergoing TIL therapy, we found that tumors that did not respond to treatment expressed significantly increased levels of *AURKA* compared to responding tumors. Both in that dataset and in an independent dataset of melanoma samples derived from patients treated with anti-PD-1 therapy, *AURKA*, *AURKB*, and *CDCA8* expression were higher in tumors that did not respond to immunotherapy. Additionally, *AURKA* and *AURKB* expression levels were significantly inversely correlated with T-cell markers in a dataset of melanoma samples derived from patients treated with anti-CTLA4 therapy. These data further strengthened the hypothesis that Aurora kinase upregulation plays a role in resistance to immunotherapy.

In our in vivo studies, the AURKB inhibitor AZD1152 enhanced the efficacy of anti-CTLA4 in vivo in the MC38/gp100 model when starting treatment in small tumors. A similar enhancement of anti-CTLA4 efficacy was observed by combination with the AURKi VX-680 (tozasertib) [44], which also yielded high comboscores in our compound screens (supplementary figure 2a, b). Furthermore, a recent study by Vilgelm et al*.*, showed that AURKA inhibition promoted TIL recruitment and enhanced the efficacy of T-cell activating immunotherapy [21]. That study used AURKA inhibitor MLN8237 (alisertib), which also gave high comboscores in our compound screens (supplementary figure 2a, b). However, tumor inhibition was reduced in more established tumors, suggesting that this specific regimen may be most efficacious against earlier-stage disease, or in the adjuvant settings of minimal residual disease. Further supporting that notion, we did not observe improved control of larger tumors in the B16, BP or D4M-UV2 melanoma models by the combination of AZD1152 combined with anti-CTLA4 or anti-PD1. Tumor growth control by anti-PD1 was not enhanced by AZD1152 either, suggesting that AZD1152 may potentiate the initiating phase of the immune response more than the effector phase, but this will need to be investigated further. In the B16 model, Aurora kinase inhibition by AZD1152 was confirmed at the transcriptional level in the tumor. Injecting i.t. had a more pronounced effect compared to i.p., despite the larger tumor sizes, and the combination effect with ACT suggests that Aurora kinase inhibition may be more effective in combination with T-cell therapy as compared to checkpoint blockade. However, Aurora kinase independent factors may limit T-cell-mediated cytotoxicity in this model with notoriously low T-cell infiltration, especially in larger tumors. Therefore, AZD1152 may be most effective in the presence of a substantial immune response, since its mechanism of action to potentiate T-cell cytotoxicity is dependent on the presence and activity of a minimal number of cytotoxic T cells in the tumor microenvironment. Although it is also possible that AURKi negatively affect immune cells in vivo, our in vitro results show no negative effects of the inhibitors on TIL viability, proliferation or cytotoxity. Blas-Rus et al. recently showed that the activation of naïve T cells is dependent on AURKA, while AURKB inhibition by AZD1152 did not affect T-cell activation [45], but the effect of AURKi on TILs has not been well studied.

To conclude, using high-throughput screens and multiple assays to study the synergy between genetic modifications or drug treatments and TILs to identify potential candidates for combination cancer treatments, we showed that Aurora kinase expression correlates with immune infiltrate, response to immunotherapy and survival in melanoma patient samples, and AURKi robustly sensitize melanoma cells to T-cell-mediated cytotoxicity in vitro and in a setting of low but not high tumor burden in vivo. These results underscore the feasibility of the tumor/TIL model system to find rational combinations with immunotherapy, as well as the justification for further studies into the role of Aurora kinases during oncogenesis. This will be crucial to understand the factors both in the tumor cells and the tumor microenvironment that determine the outcome of Aurora kinase studies in preclinical models and clinical trials.

### Electronic supplementary material

Below is the link to the electronic supplementary material.

Supplementary file1 (PDF 1869 kb)

## Data Availability

Any data and material will be shared upon request.
