# Supplementary material for: Aurora kinase inhibition sensitizes melanoma cells to T-cell-mediated cytotoxicity
Source: Cancer Immunol Immunother. 2020 Oct 29;70(4):1101–13. doi: 10.1007/s00262-020-02748-9 (PMC7979613; doi:10.1007/s00262-020-02748-9)
Supplement: Supplementary file 1 — Supplementary file1 (PDF 1869 kb) [file 262_2020_2748_MOESM1_ESM.pdf]

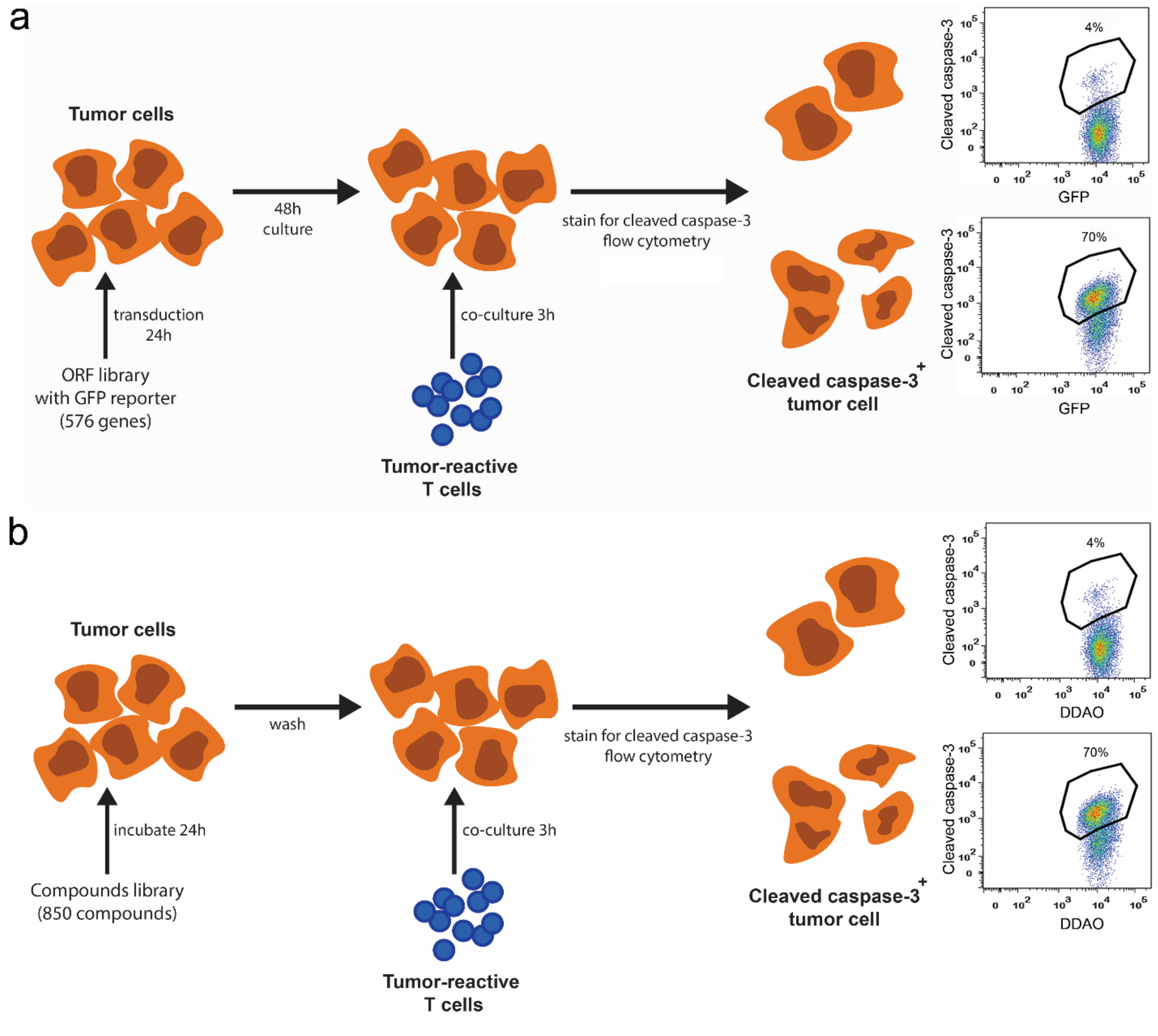

Supplementary figure 1 Methodologies high-throughput screens
(a) For the ORF screen, patient-derived melanoma tumor cells were transduced with an ORF library encoding 576 genes and a GFP reporter in an arrayed assay. After 24 hours, the medium was refreshed, and after another 48-hour cell culture, tumor cells were co-cultured with autologous TILs at a predetermined ratio (or TIL medium) for three hours, followed by intracellular staining for cleaved caspase-3. Transduced cells were selected by fluorescence-activated cell sorting of the GFP-expressing cells, followed by flow cytometry analysis of cleaved caspase-3 and calculation of the comboscores. (b) For the compound screen, patient-derived melanoma cells were labeled with the cell tracker dye DDAO and seeded in 96-well plates as 50,000 cells per well containing 1 µM of one of 850 different compounds per well in triplicate. After 24 hours, the compound was washed off, and control medium or autologous tumor-reactive TILs were added at a predetermined ratio and co-incubated for three hours. Cells were then stained for intracellular cleaved caspase-3 and the percentage of DDAO^{+}cleaved caspase-3^{+} tumor cells was used as a measure of the number of tumor cells undergoing apoptosis. For controls, DMSO-treated cells were co-cultured with autologous TILs. Additionally, the TILs were incubated with the various drugs and tested for their tumor-lysing capability to ensure that the drugs were not toxic to T cells at the concentrations used.

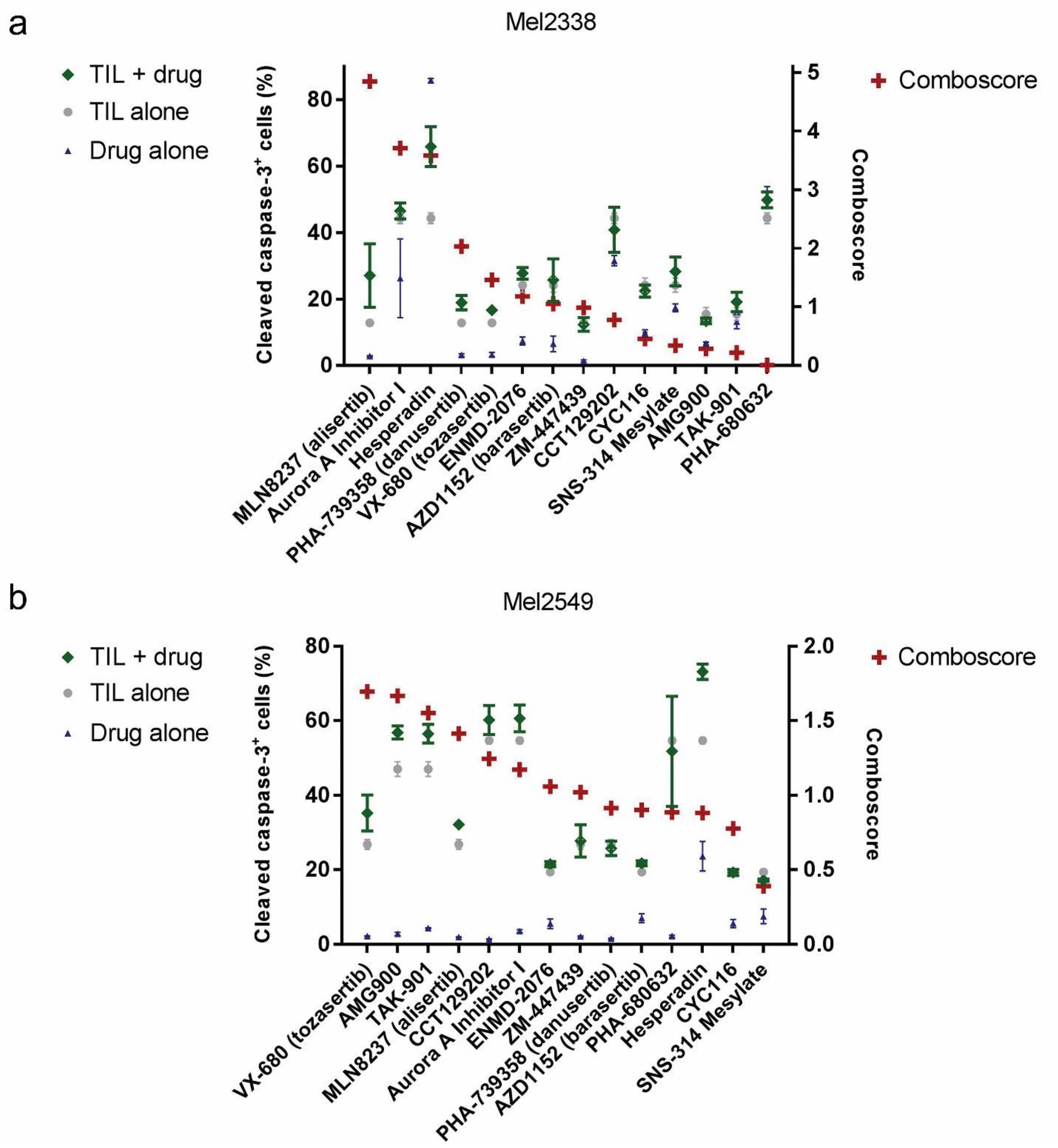

Supplementary figure 2 High-throughput compound screens
Aurora kinase inhibitors (i.e. drugs being annotated to have Aurora kinase inhibiting activity) were sorted by their individual comboscores, and comboscores (+) were plotted for the Mel2338 (a) and Mel2549 (b) cell lines, superimposed on the cleaved caspase-3 percentages induced by drug alone (Δ), TIL alone (o), and drug followed by TIL (◊). The highest comboscores indicate the most potential for synergy with immunotherapy. Since the comboscore is an absolute value, negative % caspase(drug+T cells) – % caspase(drug) values can result in high comboscores but are not of interest for this study because the TILs did not result in increased tumor cytotoxicity compared with the drug alone. In this study, Hesperadin was the only AURKi resulting in a high “negative” comboscore in the Mel2338 cell line due to a higher cell death rate induced by the drug alone than by drug + TILs.

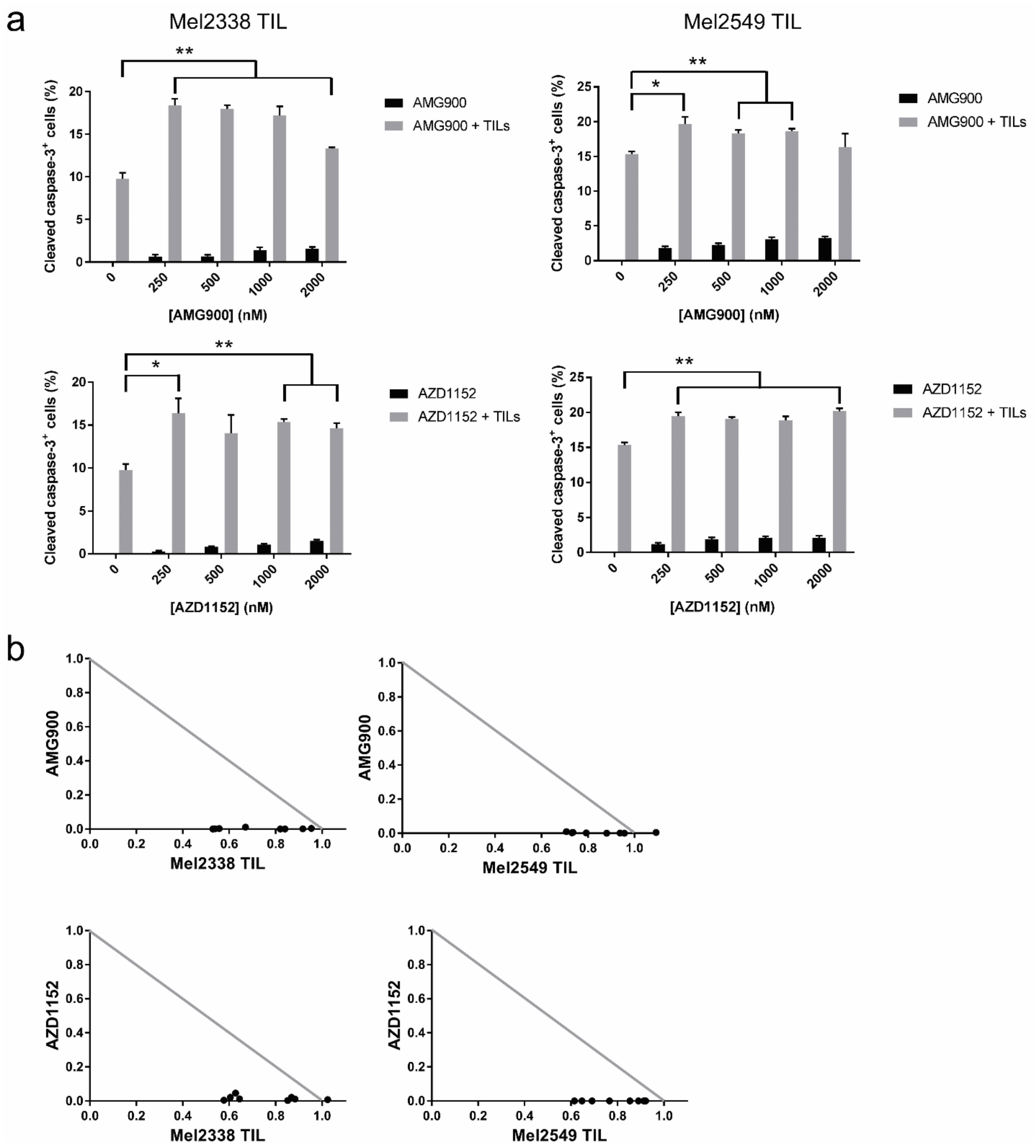

Supplementary figure 3 Aurora kinase inhibitors and TILs synergize in inducing apoptosis in human melanoma cells

(a) The human melanoma-derived cell lines Mel2338 and Mel2549 were treated with increasing concentrations of pan-Aurora kinase inhibitor AMG900 or Aurora kinase B inhibitor AZD1152 (black bars), followed by co-culture with autologous TILs (grey bars). The percentage of cleaved caspase 3 was subsequently analyzed to quantify apoptosis. (b) A combination index was calculated to quantify synergy between Aurora kinase inhibitors and melanoma-derived TILs using CalcuSyn. The combination indices of AMG900 or AZD1152 with Mel2559 or Mel2686 TIL are represented in the normalized isobolograms. The normalized dose effect of each drug is

represented on the representative axes. The combination index between the drugs is indicated in the graph by black dots, and the interaction is synergistic if the combination index is <1, below the diagonal line.

Data are representative of at least three independent experiments. Two-sided independent sample t-tests were performed to compare cleaved caspase-3^+ cell frequencies induced by a compound and TIL with TIL alone.

*$p < 0.05$

**$p < 0.01$

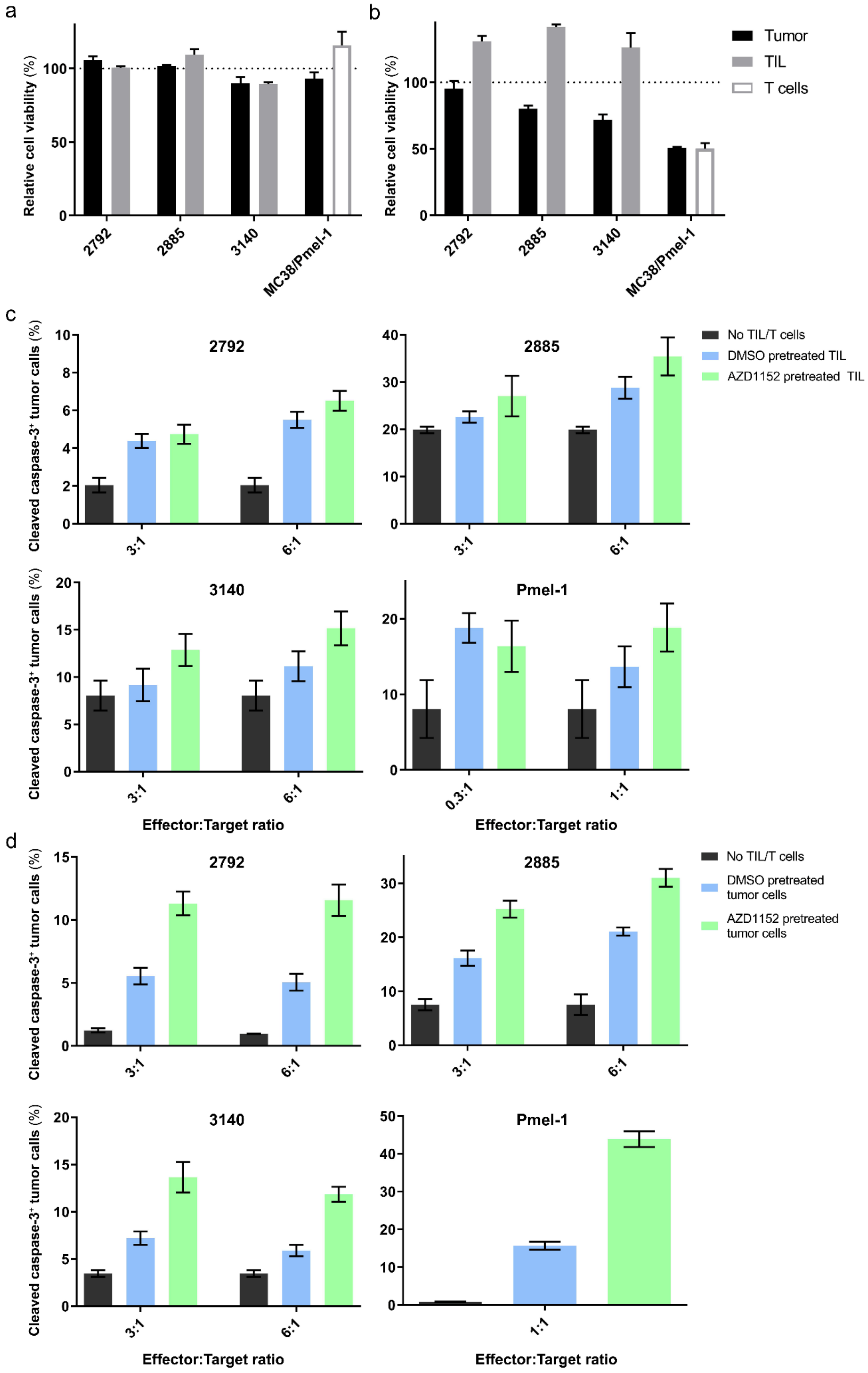

a
Relative cell viability (%)
100
50
0
2792
2885
3140
MC38/Pmel-1
b
Relative cell viability (%)
100
50
0
2792
2885
3140
MC38/Pmel-1
Tumor
TIL
T cells
c
Cleaved caspase-3⁺ tumor calls (%)
2792
2885
3140
Pmel-1
No TIL/T cells
DMSO pretreated TIL
AZD1152 pretreated TIL
3:1
6:1
0.3:1
1:1
Effector:Target ratio
d
Cleaved caspase-3⁺ tumor calls (%)
2792
2885
3140
Pmel-1
No TIL/T cells
DMSO pretreated tumor cells
AZD1152 pretreated tumor cells
3:1
6:1
1:1
Effector:Target ratio

Supplementary figure 4 Aurora kinase inhibitor AZD1152 does not decrease the viability, proliferation rate or anti-tumor cytotoxicity of TILs

The human melanoma-derived cell lines Mel2792, Mel2885, Mel3140, murine cell line MC38/gp100 (black bars) and the corresponding autologous human TIL or murine Pmel-1 T cells (grey bars) were incubated with 2µM AZD1152 or DMSO for 24 hours before performing cell viability assays using CellTiter-Blue (a) and DAPI (b). Relative cell viabilities of AZD1152 versus DMSO quadruplicate treated conditions are shown. While none of the human melanoma TIL lines tested showed a decrease in viability or proliferation, the number of viable Pmel-1 T cells did decrease based on DAPI staining (b), which could be due to a difference between human and murine mechanisms, or differences between TIL and activated splenocytes, for instance activation status. This requires further investigation. (c) The TIL or Pmel-1 T cells were incubated with 2µM AZD1152 or DMSO for 24 hours prior to co-culture with the autologous tumor cell lines and performing the cleaved caspase-3 cytotoxicity assay. (d) For reference, the cleaved caspase-3 assay was also performed after treating the matching tumor cell lines with 2µM AZD1152 or DMSO for 24 hours prior to co-culture.

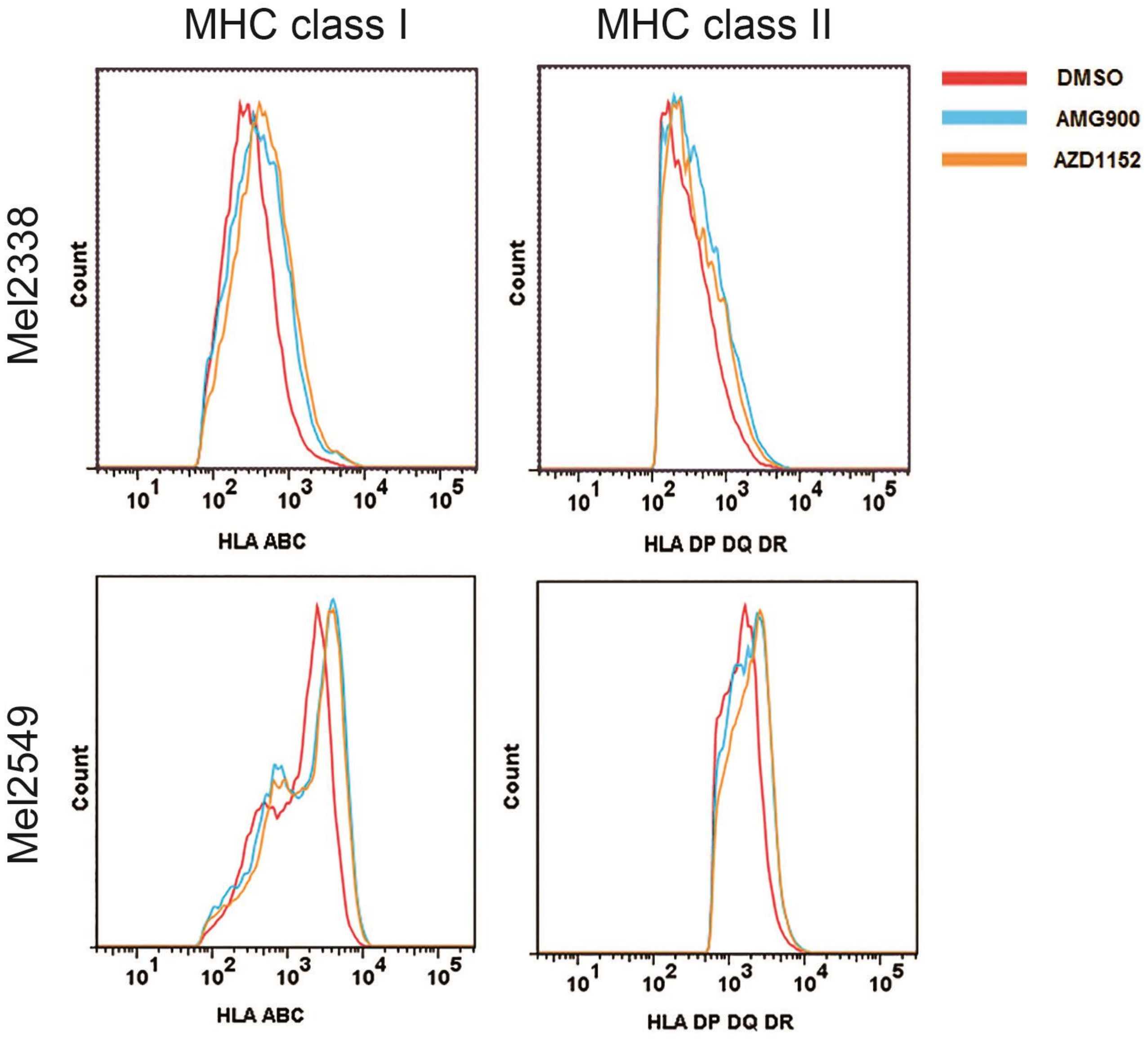

Supplementary figure 5 Effect of Aurora kinase inhibitors on human melanoma cell MHC expression

The surface expression of MHC class I and II on melanoma cell lines Mel2338 and Mel2549 was not changed to an extent that could explain the enhanced sensitization to T cell cytotoxicity by treatment with Aurora kinase inhibitors AMG900 or AZD1152 for 24 hours .

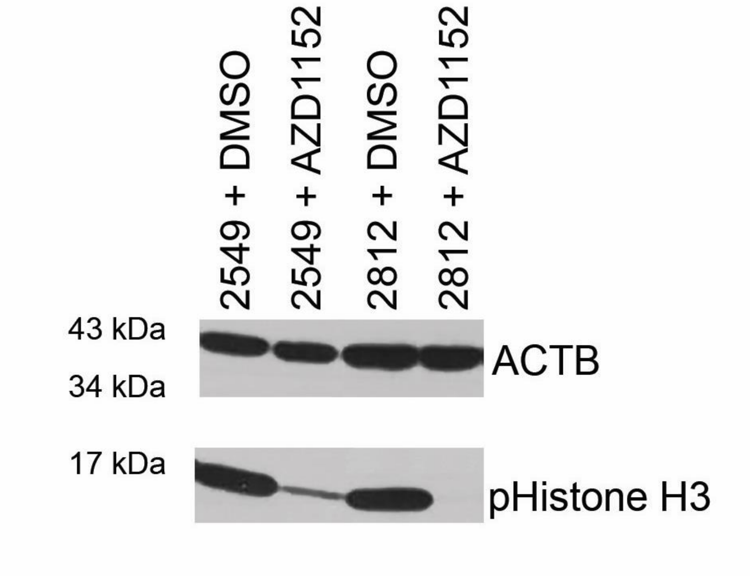

Supplementary figure 6 Aurora kinase inhibitor AZD1152 diminishes histone H3 phosphorylation

Western blot analysis of phosporylated histone H3 after treatment of Mel2549 and Mel2812 with Aurora kinase inhibitor AZD1152 or DMSO control. ACTB was used as loading control. Empty film parts have been removed, and white spaces are used between the different blot films.

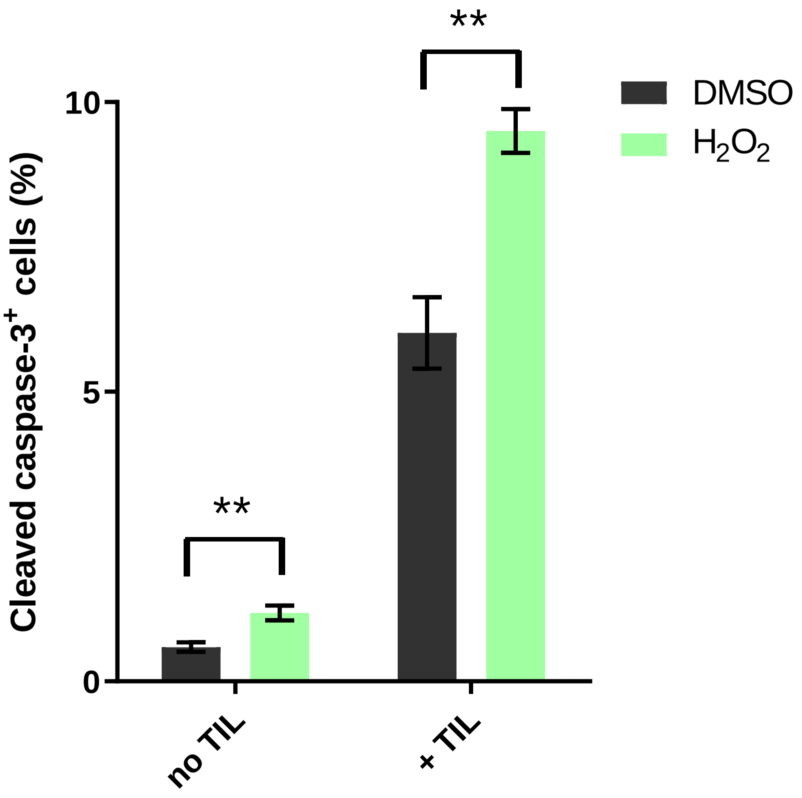

Supplementary figure 7 H_2O_2 increases sensitivity to T cell-induced cytotoxicity
Flow cytometry-based analysis of the fraction of cleaved caspase-3^+ cells after treatment of Mel2812 cells with H_2O_2 or DMSO. Two-sided independent sample t-tests were performed to compare cell frequencies between the groups.
**$p < 0.01$

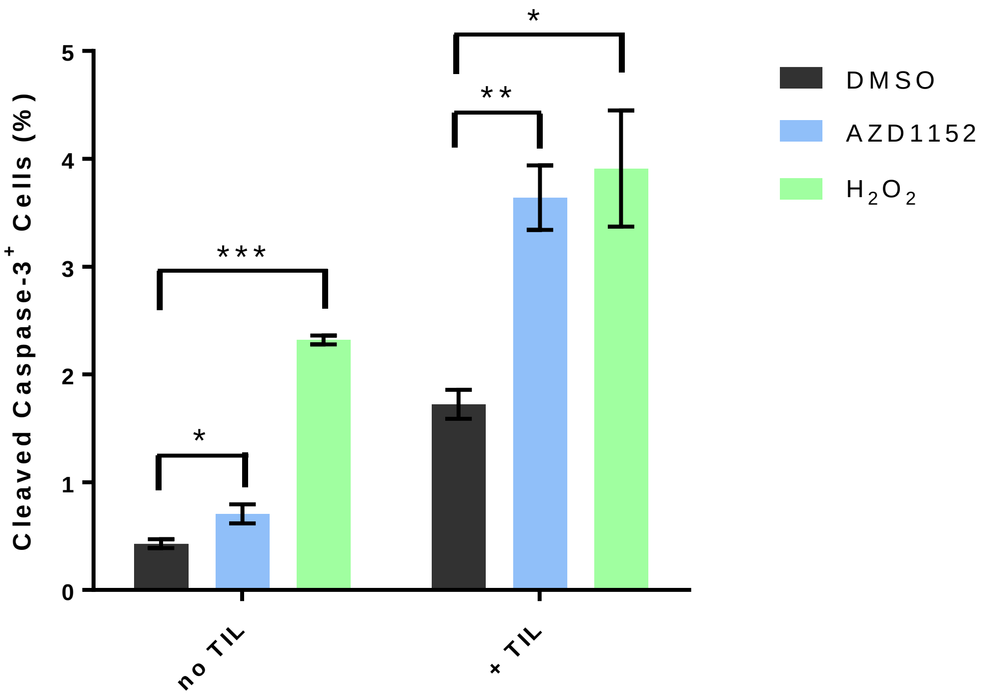

Supplementary figure 8 AZD1152-induced sensitivity to T cell-induced cytotoxicity independent of effect on proliferation

Mel2812 cells were seeded as 3×10^5 cells/well in 6-well plates and cultured overnight before treating with DMSO, AZD1152 or H_2O_2 for 24h. After drug treatments, 5×10^4 cells/well were seeded in a 96-well plate for immediate co-culture with autologous TILs to prevent potential prior drug effect on proliferation to affect cell numbers. The fraction of cleaved caspase-3^+ cells were analyzed by flow cytometry. The experiment was repeated three times with three to five biologic replicates. Differences in cell frequencies were analyzed by two-sided independent sample t-tests.

*$p < 0.05$

**$p < 0.01$

***$p < 0.001$

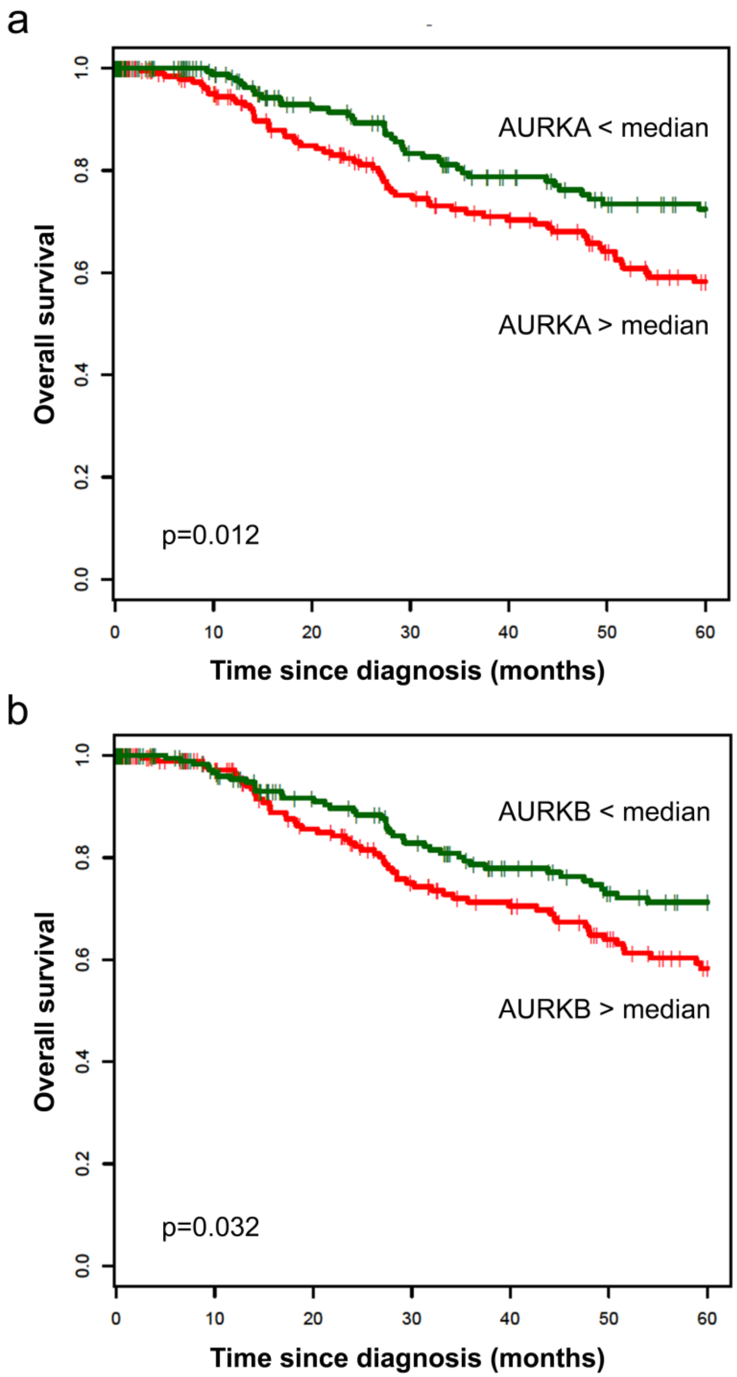

Supplementary figure 9 *AURKA* and *AURKB* expression are correlated with poor survival Kaplan-Meier 5-year survival curves for above median versus below median mRNA expression levels of *AURKA* (a) and *AURKB* (b) in the SKCM TCGA dataset (n = 459).

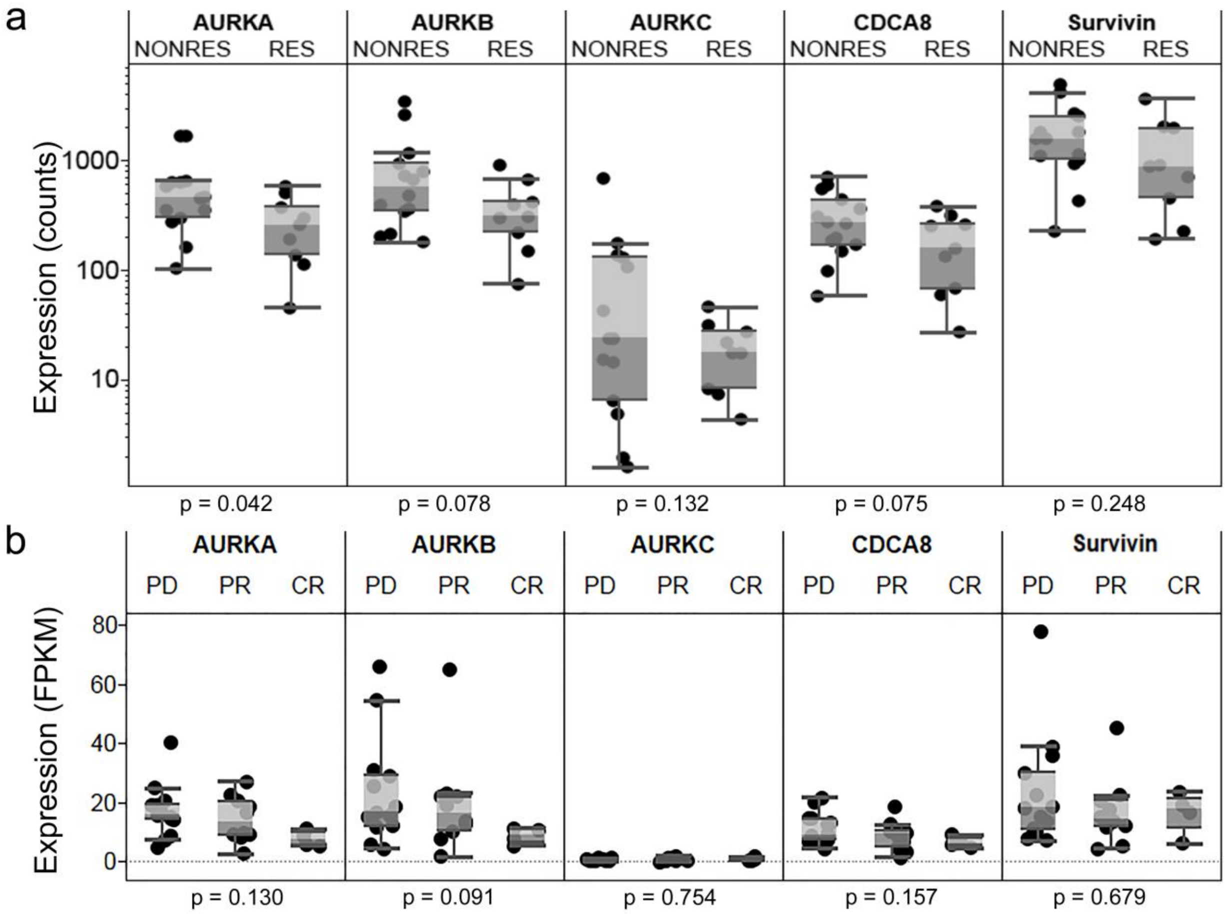

Supplementary figure 10 Aurora kinase-related expression levels in patients treated with immunotherapy

(a) Differences in *AURKA*, *AURKB*, *AURKC*, *CDCA8*, and Survivin normalized expression in melanoma samples from patients who did (RES = responders; n = 9) or did not (NONRES = non-responders; n = 14) respond to TIL therapy, analyzed using NanoString. The bars indicate the distance between the interquartile range and the maximum upper or lower value, excluding outliers. Expression levels between responding and non-responding patients were compared by Mann-Whitney U tests. (b) Expression levels of *AURKA*, *AURKB*, *AURKC*, *CDCA8*, and Survivin in melanoma samples of patients with progressive disease (PD; n = 13), partial response (PR; n = 10), or complete response (CR; n = 4) to anti-PD-1 therapy. These RNA sequencing data were obtained from a previously published dataset in which none of the tumors had been characterized as stable disease. Kruskal-Wallis non-parametric tests were used to compare the expression levels between the three groups.

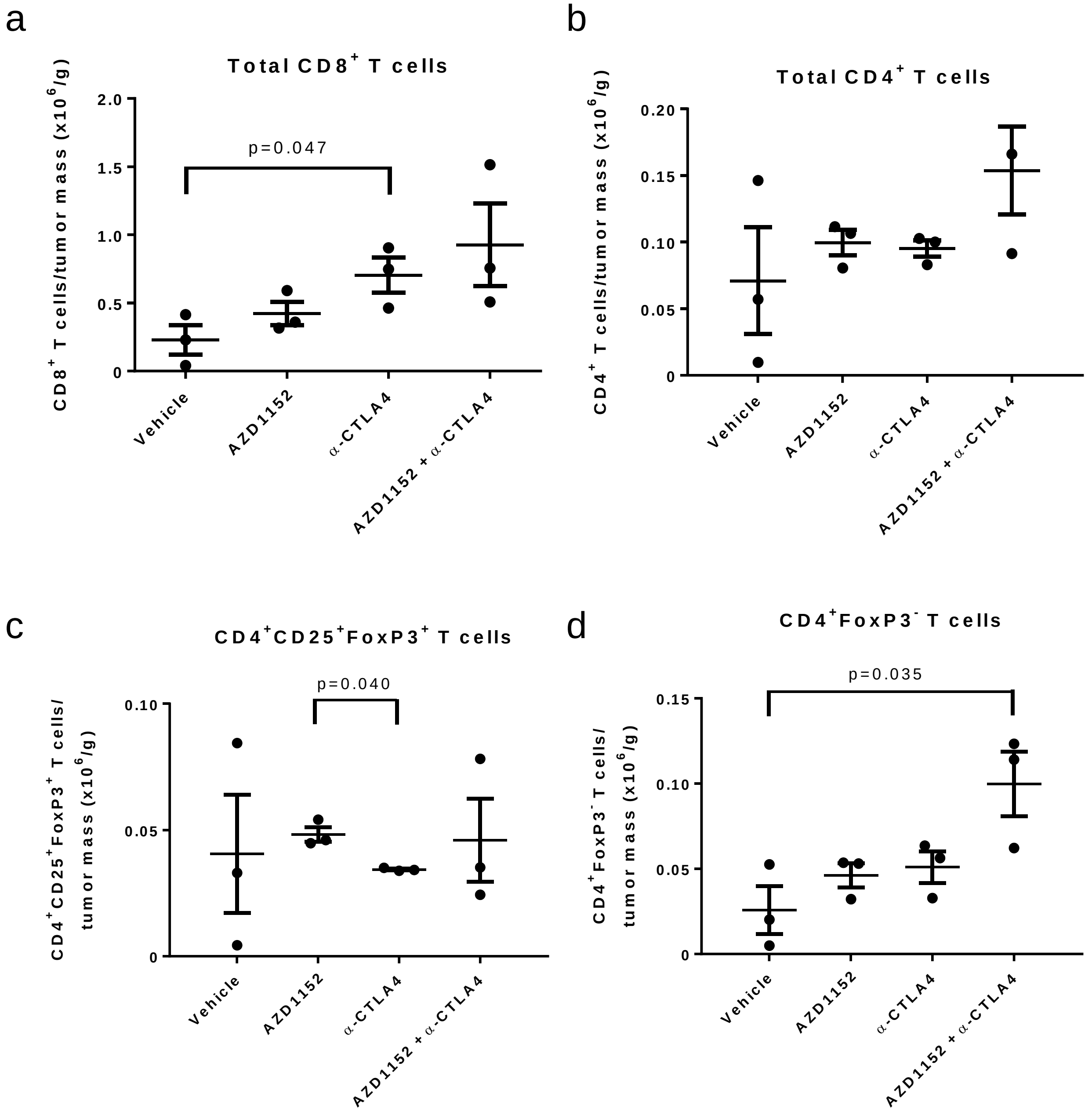

Supplementary figure 11 Effects of Aurora kinase inhibitors AZD1152 and anti-CTLA4 on T cell populations

Tumor-infiltrating immune cells were harvested from tumors on day 18. Cells were first gated for $CD45^+CD3^+$ T cells and subsequently analyzed for T cell subpopulations. No significant effects of AZD1152 on the number of $CD8^+$ cytotoxic T cells (a), $CD4^+$ T helper cells (b), $CD4^+CD25^+FoxP3^+$ regulatory T cells (c), or effector $CD4^+$ T cells (d) relative to tumor volume were observed, although the tumors treated with AZD1152 seemed to have slightly more regulatory T cells compared with the anti-CTLA4-treated group (shown in c). Two-sided independent sample t-tests were performed to compare cell frequencies between the groups.

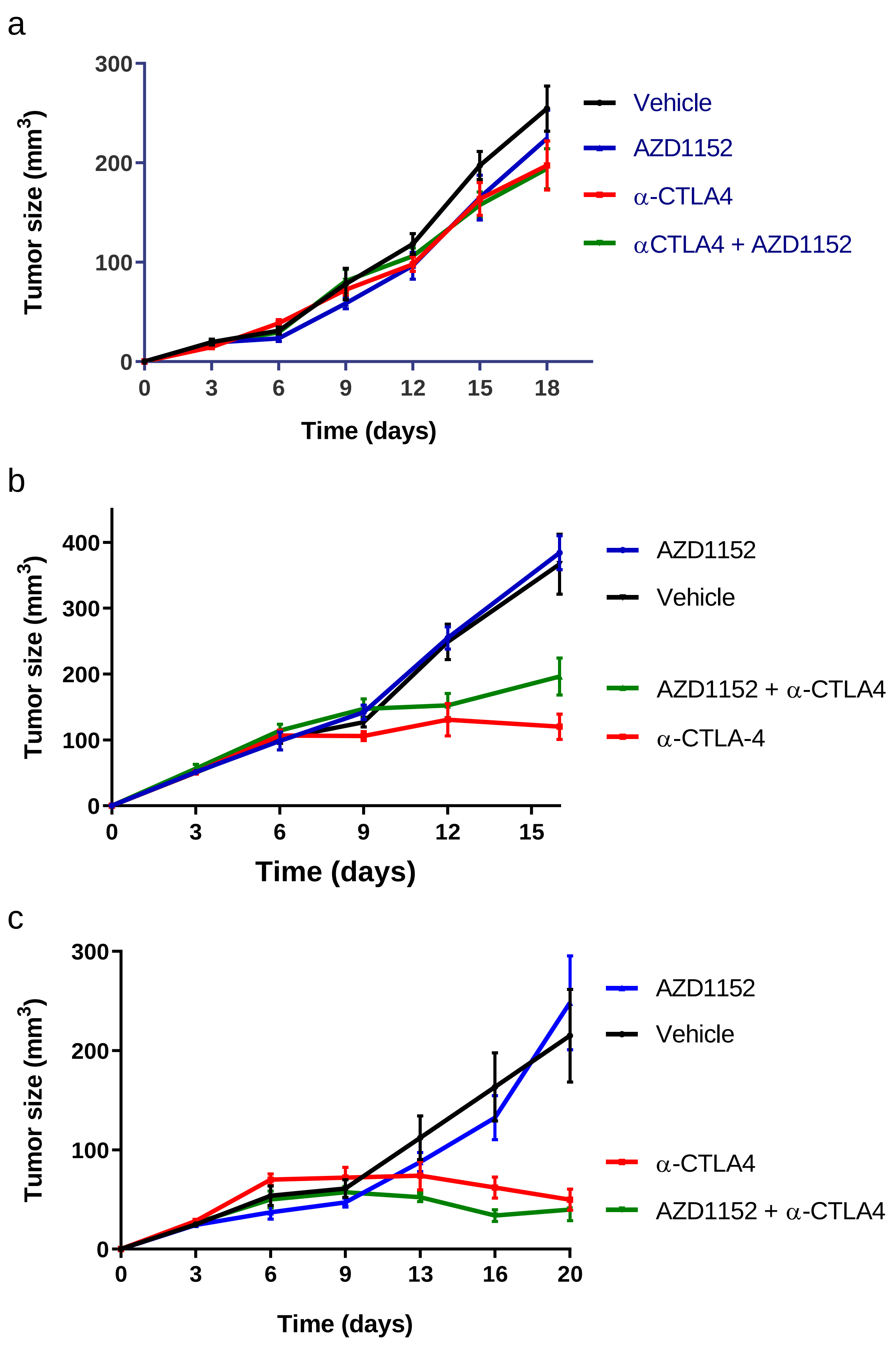

Supplementary figure 12 Aurora kinase B inhibitor in combination with anti-CTLA4 treatment in MC38/gp100, BP and D4M UV2 tumor models

(a) Repeating the experiment shown in fig. 4a with larger tumors (avg tumor size at treatment initiation: 15 mm^2). (b) The same experiment starting treatments on day 6 after inoculation of 1.0×10^6 BP cells (avg tumor size: 38 mm^2), and using 25 µg anti-CTLA4 and 5 mg/kg AZD1152. (c) The same experiment in the D4M UV2 melanoma model, using 50 µg anti-CTLA4 (avg tumor size at treatment initiation: 17 mm^2).

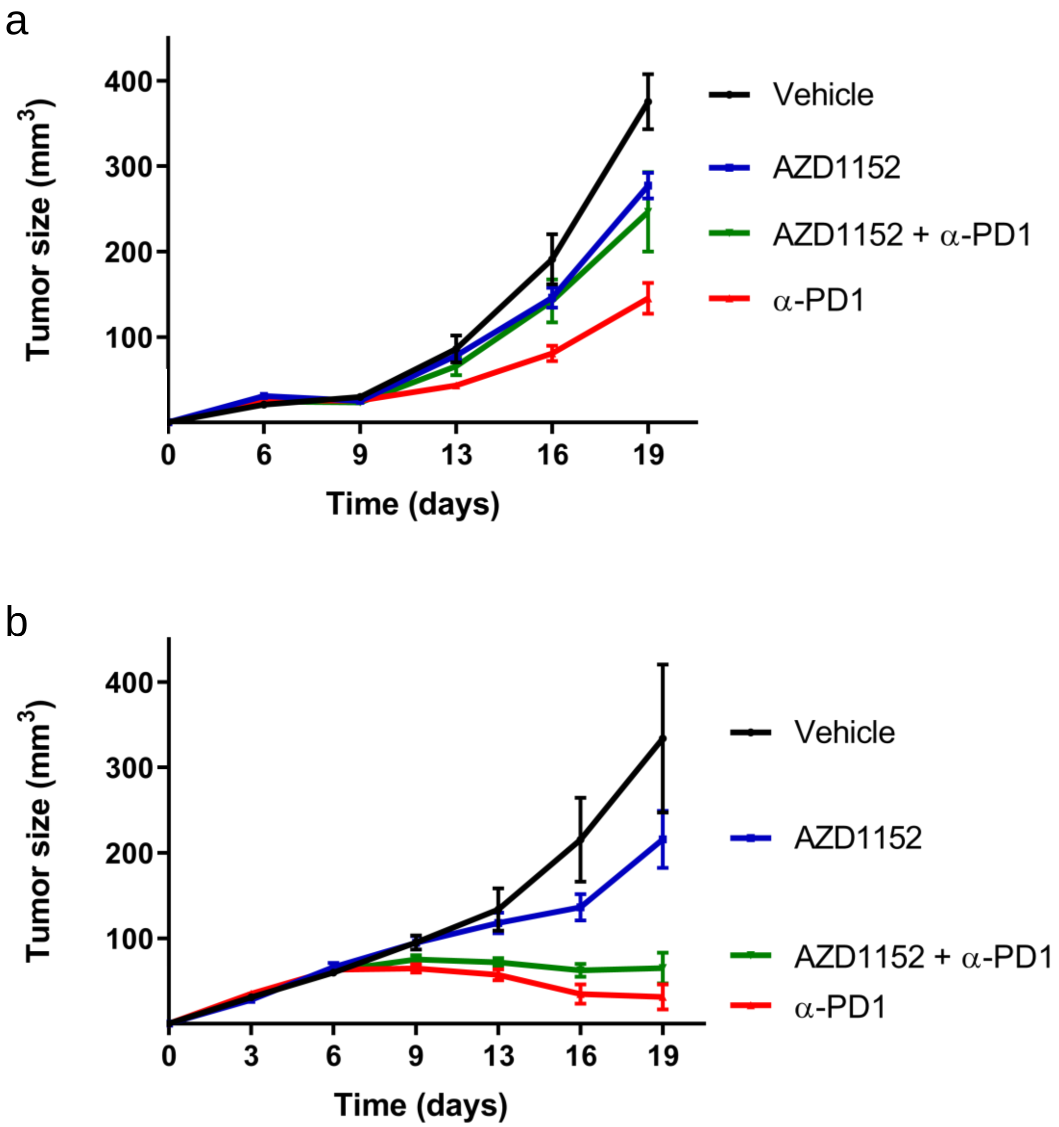

Supplementary figure 13 Aurora kinase B inhibitor in combination with anti-PD1 treatment in MC38/gp100 and D4M UV2 tumor models

(a) Tumor growth curves after inoculating 0.5×10^6 MC38/gp100 cells and starting treatment three days post tumor cell injection (avg tumor size <15 mm^2) of 25 mg/kg AZD1152 for four days and 150 µg anti-PD1 every three days. (b) Tumor growth curves after injecting 0.5×10^6 D4M UV2 cells and starting treatment on day three (avg tumor size: 17 mm^2) of 25 mg/kg AZD1152 for four days and 25 µg anti-PD1 every three days.

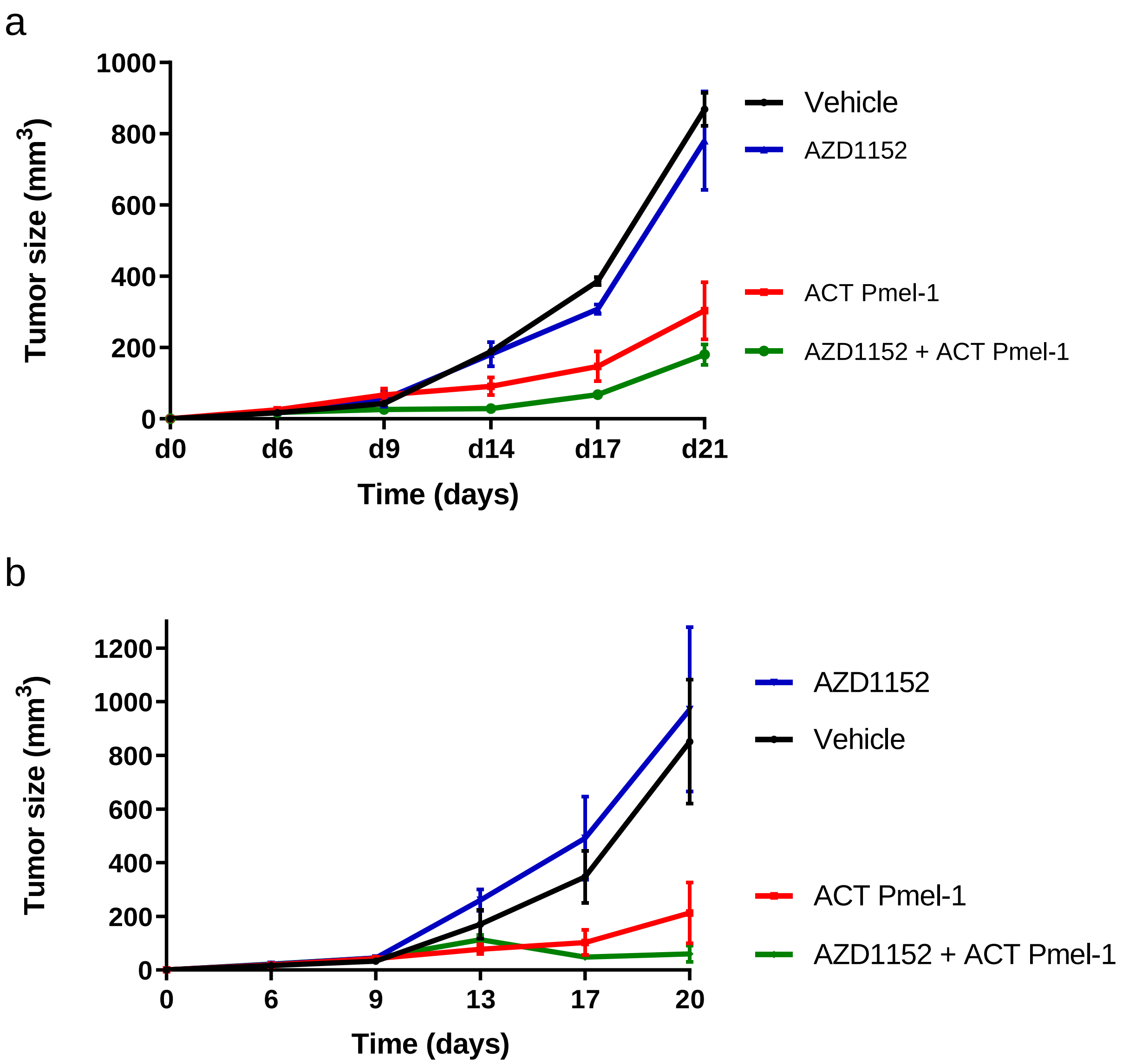

Supplementary figure 14 Aurora kinase B inhibitor treatment in combination with Pmel-1 ACT in B16 tumor model

(a) AZD1152 was administered intraperitoneally on days 3-6, and Pmel-1 T cells (1×10^6) were adoptively transferred on day 7 post tumor injection (n=3-4/group; avg tumor size day 3: 15 mm^2). (b) Pmel-1 T cells (0.6×10^6) were adoptively transferred on day 7 and AZD1152 was administered intratumorally on days 10-13 post tumor injection (n=5-6/group; avg tumor size day 10: 26 mm^2). Measurements are a reflection of tumor or resulting wound or scar sizes. Mice receiving ACT alone were sacrificed predominantly due to tumor size, whereas mice treated with the combination of AZD1152 and ACT were predominantly sacrificed due to wound formation or ulceration, not tumor size.
